# Total Synthesis of Peniterphenyls A and E

**DOI:** 10.3390/md23110437

**Published:** 2025-11-14

**Authors:** Huayan Xu, Yuyue Li, Yuecheng Fang, Juan Liu, Junfeng Wang, Shengrong Liao, Yonghong Liu

**Affiliations:** 1 Wuya College of Innovation, Shenyang Pharmaceutical University, Shenyang 110016, China; 2 Research Center for Marine Microbes, Laboratory of Tropical Marine Bio-Resources and Ecology, Guangdong Key Laboratory of Marine Materia Medica, South China Sea Institute of Oceanology, Chinese Academy of Sciences, Guangzhou 510301, China; 3 University of Chinese Academy of Sciences, Beijing 100049, China

**Keywords:** *p*-terphenyls, total synthesis, site-selectivity, cross-coupling, S_N_Ar, Pd catalysis

## Abstract

Our previously discovered marine natural products, peniterphenyls A and E, exhibit superior anti-herpes simplex virus 1/2 (HSV 1/2) activity, probably via interference with virus adsorption and membrane fusion to host cells. Their clear mechanism mode still remains unresolved due to its limited availability from nature. This study establishes their first site-selective chemical total syntheses, affording peniterphenyls A and E in overall yields of 4.5% (over thirteen steps) and 2.3% (over twelve steps), respectively. A nucleophilic aromatic substitution (S_N_Ar) between compounds **4** and **5**, and a direct C(sp^2^)–H/C(sp^2^)–H oxidative coupling using the Pd(TFA)_2_/AgOAc catalyst system with a pivaloyl directing group conveniently furnishes the dibenzofuran core with good efficiency. Steric hindrance and substituent directing effects of arene govern the high site-selectivity of the Pd-catalyzed C(sp^2^)–H activation during furan formation. Featuring readily available materials and straightforward operations, this synthetic route provides convenient access to these bioactive natural products for further study.

## 1. Introduction

**Figure 1:**
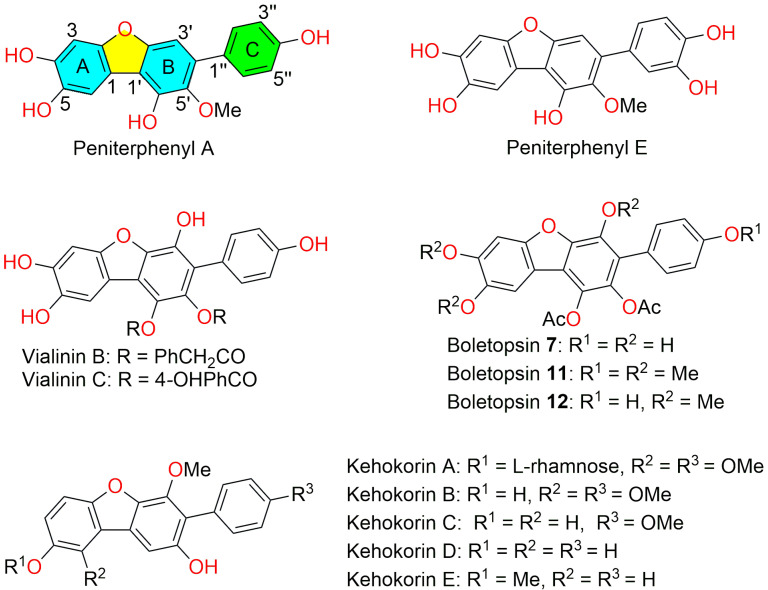
Peniterpehenyls A, E and the totally synthesized natural dibenzofuranoid *p*-terphenyls.

*p*-Terphenyl natural products represent a highly oxygenated family of compounds characterized by a linear three-benzene-ring backbone. These compounds exhibit diverse biological activities, including cytotoxicity, antimicrobial activity, and inhibition of neuraminidase and phospholipase A2 (PLA2) [1,2]. Peniterphenyls A and E belong to this family while featuring a distinctive 3-phenyldibenzofuran core structure (Figure 1).

Peniterphenyl A was first discovered by our group from the deep-sea-derived fungus *Penicillium* sp. SCSIO41030 [3]. Peniterphenyl E (originally reported as 3′-demethoxy-6′-desmethyl-5′-methoxycandidusin B) was initially isolated by Belofsky and colleagues [4], and later also identified in our Laboratory [3]. Structurally, peniterphenyl E differs from peniterphenyl A by an additional hydroxyl group at the C3″ position of the C4′-phenyl unit (Figure 1). Both compounds exhibit promising anti-herpes simplex virus 1/2 (HSV-1/2) activity, with EC_50_ values of 2.2/1.4 μM for peniterphenyl A and 4.3/2.4 μM for peniterphenyl E, demonstrating potency comparable to or slightly superior to that of the clinical drug acyclovir (EC_50_ = 3.6/4.6 μM). Preliminary mechanistic studies indicated that peniterphenyl A interferes with HSV-1/2 viral entry by potentially binding to the viral envelope glycoprotein D, thereby disrupting virus adsorption and membrane fusion. However, the clear mode of action and inhibition mechanism remains elusive due to their limited natural availability.

Given their intriguing structures, notable medicinal properties, and the critical requirement of peniterphenyl A for our ongoing mechanistic studies on anti-HSV1/2 activity, the development of an efficient synthetic methodology is imperative.

**Scheme 1:**
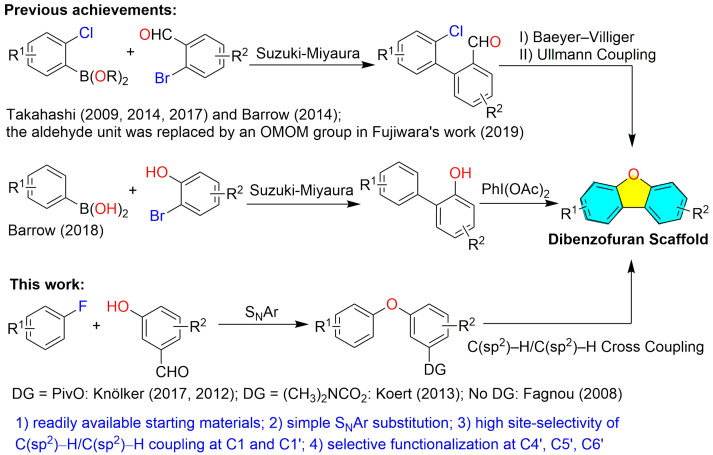
Strategies for constructing the dibenzofuran core in the total synthesis of *p*-terphenyl derivatives.

Notably, several previous studies have achieved the total syntheses of dibenzofuran-containing *p*-terphenyls (Figure 1), including vialinin B [5] and C [6], boletopsins **7**, **11**, and **12** [7], as well as kehokorins A–E [8,9]. A primary synthetic challenge in constructing these unique molecules lies in the assembly of their highly oxygenated dibenzofuran core (Scheme 1). To achieve site-selective furan ring formation, Takahashi [5,6,8], Barrow [7], and Fujiwara [9] developed strategies involving pre-functionalization of the four key positions with boronic acid, halogen (Cl, Br), and the aldehyde group or methoxymethoxy (OMOM) moiety. The dibenzofuran scaffold was then assembled through sequential Suzuki–Miyaura cross-couplings, Baeyer–Villiger oxidation, and intramolecular Ullmann coupling. Later, Barrow and co-workers [10] constructed the diphenyl fragment via Suzuki–Miyaura coupling and prepared the dibenzofuran core through an oxidative cyclization using PhI(OAc)_2_. In contrast, Knölker [11,12], Fagnou [13], and Koert [14] reported Pd-catalyzed-directed C(sp^2^)–H/C(sp^2^)–H oxidative coupling strategies for dibenzofuran synthesis, though some cases suffered from site-selectivity limitations [11,14,15]. Interestingly, despite its potential, this strategy has not yet been applied to the synthesis of *p*-terphenyl-type dibenzofurans, likely due to persistent challenges in regiocontrol.

Based on these advances, we herein describe a streamlined approach to dibenzofuran assembly via a simple and operationally convenient Pd(TFA)_2_/AgOAc-catalyzed C(sp^2^)–H/C(sp^2^)–H oxidative coupling. This strategy enabled the first total syntheses of peniterphenyls A and E from commercially available starting materials (2,5-dihydroxybenzaldehyde and 2-(benzyloxy)-4-fluorobenzaldehyde) in thirteen and twelve steps, respectively. Key advantages of our route include (1) controlled functionalization around the dibenzofuran scaffold (C4′-arylation, C5′-*O*-methylation, and C6′-hydroxylation) through the synthesis of the key intermediate **4** [16]; (2) highly site-selective C1–C1′ cross coupling achieved via C(sp^2^)–H activation, leveraging substituent directing effects of arene ring and the steric hindrance; (3) a nucleophilic aromatic substitution (S_N_Ar) strategy replacing the traditional Ullmann reaction for diphenyl ether formation; and (4) eliminating unique arylboronic acid precursor preparation.

## 2. Results and Discussion

### 2.1. Retrosynthetic Analysis for Peniterphenyl A

**Scheme 2:**
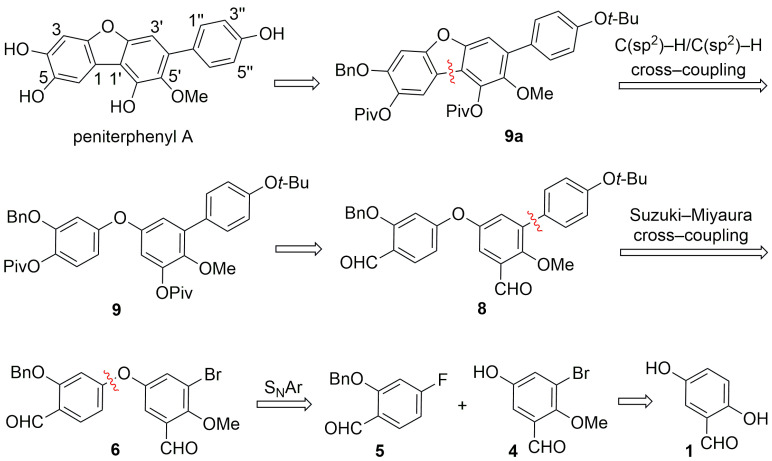
Retrosynthetic analysis for peniterphenyl A.

**Scheme 3:**
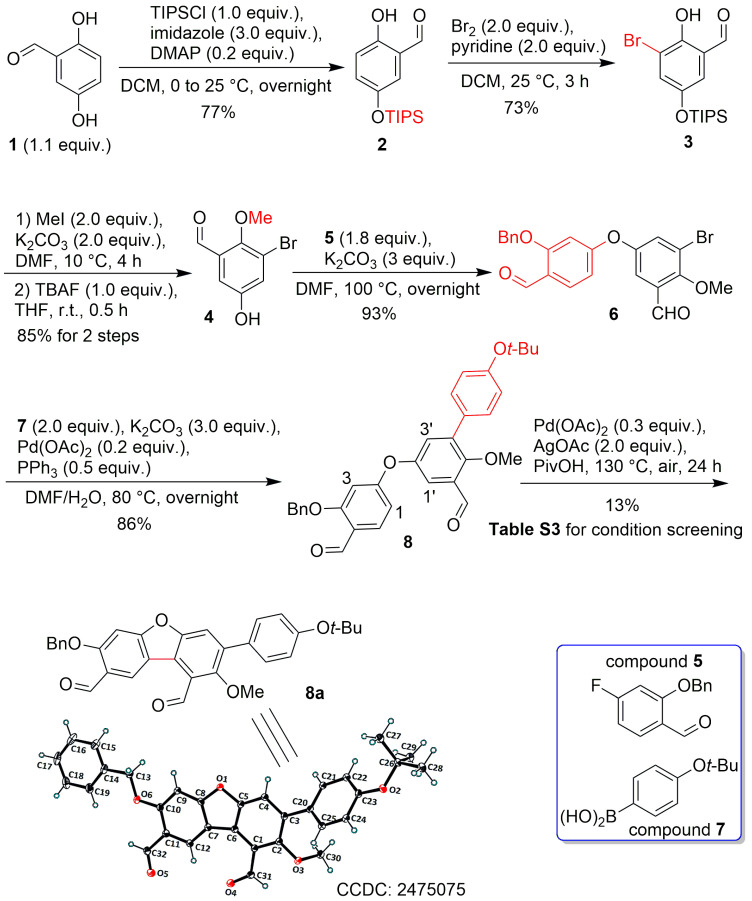
Synthesis of compound **8a**.

Our retrosynthetic analysis (Scheme 2) proposes that peniterphenyl A can be accessed through global deprotection of intermediate **9a**. Disconnection of the furan ring in compound **9a** at the C1–C1′ bond suggests that its precursor **9** can be synthesized from **8** via Baeyer–Villiger oxidation, followed by hydrolysis and subsequent acylation with pivaloyl chloride (PivCl). Compound **8** is envisioned to arise from a Suzuki–Miyaura cross-coupling between phenylboronic acid **7** (Scheme 3) and the diphenyl ether **6**. The latter can be prepared through an S_N_Ar between **5** and **4**. Compound **4** is synthesized in four conventional steps from commercially available 2,5-dihydroxybenzaldehyde (**1**). Notably, the same synthetic sequence can be applied to access peniterphenyl E by replacing boronic acid **7** with (3,4-bis(benzyloxy)phenyl)boronic acid and performing the Suzuki–Miyaura coupling with **6** under similar conditions.

### 2.2. Synthesis of Compound ***8a***

The synthesis began with selective *O*-silylation at the C5-position of commercially available 2,5-dihydroxybenzaldehyde (**1**) using triisopropylsilyl chloride (TIPSCl), which afforded compound **2** (Scheme 3). Subsequent regioselective bromination of **2** with bromine yielded **3**, which was then subjected to methylation and desilylation to furnish the key intermediate **4** in 85% yield over two steps. It is noteworthy that anhydrous DMF was essential during the *O*-methylation step to prevent *O*-desilylation and avoid undesired 2,5-*O*-dimethylation. Achieving three distinct site-selective transformations—C5′-*O*-methylation, C4′-arylation, and C6′-hydroxylation—posed a significant challenge. Therefore, compound **4** served as a pivotal intermediate, enabling precise functionalization at these positions during peniterphenyls preparation. With **4** in hand, the diphenyl ether **6** was synthesized via an S_N_Ar reaction between **4** and compound **5**, employing K_2_CO_3_ as base in DMF at 100 °C. In comparison to Ullmann-type couplings, this S_N_Ar strategy offers operational simplicity and avoids metal residue in the product [17]. A Suzuki–Miyaura coupling was then carried out between phenylboronic acid **7** and compound **6** to afford compound **8**. This reaction sequence offers two advantages: it increases steric hindrance to facilitate a high site-selectivity in the forthcoming cyclization at the C1′-position and prevents debromination during the furan formation step.

At this stage, attempts to cyclize compound **8** to form **8a** (Scheme 3) under various conditions (Table S3, see Supporting Information) based on literature procedures [18] proved inefficient, affording a maximum yield of only 16% (entry 2, Table S3). Delightedly, X-ray crystallographic analysis confirmed that **8a** was indeed the desired product (Scheme 3), cyclized at the correct positions (e.g., C–C bond formed between C1 and C1′, not between C1 and C3′, C1′ and C3, or C3 and C3′, Scheme 3), with almost no site-isomers detected. These results demonstrate successful site-selective furan formation. The observed regiocontrol is likely attributable to the substituent directing effect and the steric hindrance of the benzyloxy group on ring A, inducing oxidative coupling at the C1-position; the steric hindrance from ring C directs the second coupling site at the C1′-position.

### 2.3. Condition Optimization for the Synthesis of Compound ***9a***

In consideration of the possible significant impact of the directing group on this cyclization, we replaced the formyl unit with a pivalate (PivO, in compound **9**) or an *N*,*N*-dimethylcarbamate group ((CH_3_)_2_NCO_2_, in compound **10**) and evaluated the cyclization conditions (Table 1). Pleasingly, the use of the pivalate directing group with Pd(OAc)_2_ (0.3 equiv.) and AgOAc (2.0 equiv.) significantly improved the yield to 53% (entry 1, Table 1), while reducing the amount of remaining starting material (7%) compared to the formyl-directing system. Increasing the AgOAc loading to 3.0 equiv. and raising the temperature to 160 °C resulted in complete consumption of the starting material but a lower yield of 46% (entry 2). Further increasing AgOAc amount (entry 3) or conducting the reaction under a nitrogen atmosphere (entry 4) led to markedly diminished yields despite full conversion, suggesting potential over-oxidation of starting material and/or product. Unsurprisingly, the control experiment with no AgOAc resulted in a low yield (entry 5), confirming its essential role. These findings highlight the critical influence of both the pivalate directing group and AgOAc on reaction efficiency. The electron-rich nature of **9** and **9a** makes them susceptible to decomposition in the presence of AgOAc [19]; thus, a balanced amount of AgOAc must be recruited between facilitating Pd (0)-catalyst reoxidation and preventing substrate/product degradation. Notably, replacing Pd(OAc)_2_ with Pd(TFA)_2_ slightly increased the yield (entry 6). The *N*,*N*-dimethylcarbamate group exhibited comparable efficacy in promoting the cyclization (entries 7–9). Given that pivalic acid (PivOH) proved to be a superior solvent for the reaction (entries 1–8, Table S4), the pivalate group was therefore chosen as the directing group for further optimization. Subsequent screening of other silver additives (entries 9–15, Table S4), palladium catalysts (entries 16–22), elevated loadings of AgOAc (entries 23–24) or Pd(TFA)_2_ (entries 25–26), and extended reaction time (entry 27) did not yield further improvements. Other variations (entries 28–33) were also unsuccessful. Consequently, the optimal conditions were established as follows: Pd(TFA)_2_ (0.3 equiv.), AgOAc (2.0 equiv.), pivalate as the directing group, in PivOH at 130 °C for 10 h, affording the cyclic product **9a** in 60% NMR yield (71% based on recovery of the starting material (brsm)) or 53% isolated yield (60% brsm). This key cyclization step was successfully scaled to 0.73 mmol, providing **9a** in 41% isolated yield (45% brsm).

### 2.4. Proposed Mechanism for the Pd(TFA)_2_/AgOAc-Catalyzed C(sp^2^)–H/C(sp^2^)–H Coupling

**Figure 2:**
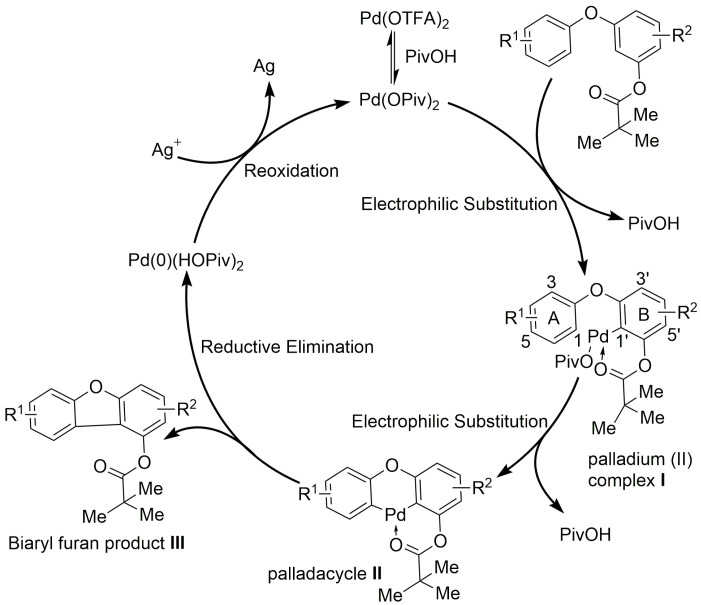
Proposed mechanism for dibenzofuran scaffold formation catalyzed by Pd(TFA)_2_/AgOAc.

Previous studies have disclosed a similar mechanism for this cyclization step [12]. The process begins with the coordination of Pd(TFA)_2_ (or the in situ formed Pd(OPiv)_2_) to the pivalate group, followed by intramolecular electrophilic palladation to generate palladium(II) complex **I**. This complex then undergoes a second C–H bond activation—cyclometallation—at the sterically less hindered positions. Specifically, the C3′ position is shielded by the proximal C4′-benzene ring, while on the A ring, the combined steric bulk of the benzyloxyl (BnO) group (at C4) and inherent directing effects favor activation at C1 over C3. This site-selectivity leads to the formation of palladacycle **II**. Subsequent reductive elimination from **II** forges the C–C bond, yielding the biaryl furan product **III** and a palladium(0) species. To close the catalytic cycle, the palladium(0) species is reoxidized to palladium(II) by a silver(I) salt (Figure 2)

### 2.5. Final Syntheses of Peniterpheynyls A and E

**Scheme 4:**
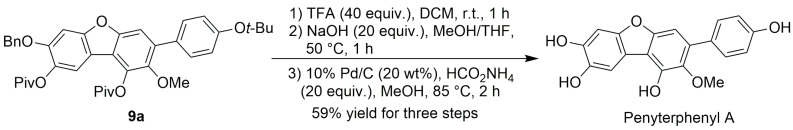
Preparation of peniterphenyl A.

**Scheme 5:**
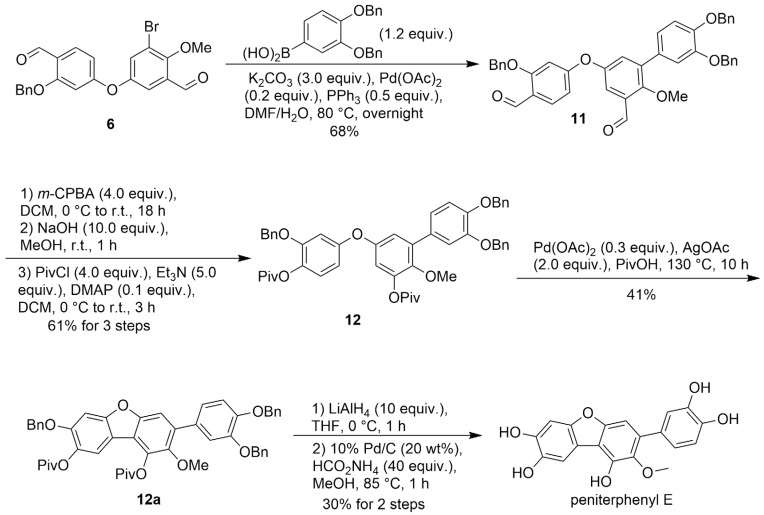
Preparation of peniterphenyl E.

Following the synthesis of compound **9a**, peniterphenyl A was obtained through a three-step sequential deprotection sequence: first, removal of the *t*-Bu with TFA; then hydrolysis of the pivalate ester under NaOH; and finally, debenzylation using HCO_2_NH_4_ and 10% Pd/C. This sequence afforded the target natural product in 59% yield over the three steps (Scheme 4). It is worth noting that first attempting debenzylation led to unintended hydrolysis of one pivalate group (as monitored by HRMS), resulting in an unstable intermediate that hampered further deprotections. These observations underscore the critical importance of the reaction sequence in this multistep process. Peniterphenyl A was thus synthesized in an overall yield of 4.5% over thirteen steps. In an alternative route to peniterphenyl E, 4-*tert*-butoxybenzoboronic acid (**7**) was replaced with 3,4-dibenzyloxybenzoboronic acid. Due to the low solubility of compound **12a** in MeOH or MeOH/THF solvent system, subsequent removal of the pivalate group using LiAlH_4_ in THF [20], followed by similar reaction conditions and procedures (Scheme 5), afforded peniterphenyl E in an overall yield of 2.3% over twelve steps. It should be mentioned that peniterphenyl E exhibited significant instability under common purification conditions. It proved highly susceptible to degradation during conventional silica gel flash column chromatography, resulting in negligible recovery. Furthermore, even when purified by HPLC, the compound required careful and rapid handling under an inert N_2_ atmosphere during solvent removal to prevent decomposition.

The structures of both synthetic peniterphenyls A (Table S1, Figures S1, S2, and S5) and E (Table S2, Figures S3, S4, and S6) were unambiguously confirmed through spectroscopic analysis (^1^H/^13^C NMR and HRMS). The operational simplicity of this route highlights its significant synthetic utility for the preparation of these biologically active natural products.

## 3. Materials and Methods

### 3.1. General Methods

Anhydrous *N*,*N*′-dimethylformamide (DMF) and other analytic grade solvents were purchased from commercial suppliers and used without further purification. Dichloromethane (DCM) was distilled over CaH_2_. Pd catalysts, Ag additives, and other chemicals were obtained from commercial sources without further purification. Mass spectrometry data were collected with a Bruker amaZon SL (Rheinstetten, Baden-Württemberg, Germany) instrument for low-resolution or a Bruker maXis/Q-TOF (Rheinstetten, Baden-Württemberg, Germany) instrument for high-resolution via ESI ionization. The NMR spectra were recorded on a 500 or 700 Hz spectrometer with TMS as an internal standard. The residual solvent peaks were used for the chemical shifts as an internal reference (ppm): ^1^H (CDCl_3_: *δ* 7.26, CD_3_OD: *δ* 3.31, (CD_3_)_2_SO: 2.50, CD_2_Cl_2_: 5.32); ^13^C (CDCl_3_: *δ* 77.0, CD_3_OD: *δ* 49.0, (CD_3_)_2_SO: 39.5, CD_2_Cl_2_: 54.0). Flash column chromatography was performed using silica gel (200–300 mesh). Thin-layer chromatography (TLC) analyses were performed on HSGF254 plates, which were visualized using UV light (λ = 254 nm).

### 3.2. Synthesis of Compounds

#### 3.2.1. Synthesis of 2-Hydroxy-5-((Triisopropylsilyl)Oxy)Benzaldehyde (**2**)

In a 50 mL flask containing 2,5-dihydrobenzaldehyde (**1**, 20.0 mmol, 2.76 g, 1.1 equiv.), DMAP (3.64 mmol, 444 mg, 0.2 equiv.), and imidazole (54.6 mmol, 3.71 g, 3.0 equiv.) dissolved in dry DCM (20 mL), TIPSCl (18.8 mmol, 3.89 mL, 1.0 equiv.) was added dropwise at 0 °C, and the reaction was stirred overnight at this temperature. After the reaction was completed, additional DCM (20 mL) was added, and the solution was washed with water (10 mL × 3). The organic layer was dried over anhydrous MgSO_4_, removed under reduced pressure, and the residue was purified with silica gel chromatography (eluent: PE/AcOEt = 50/1) to afford compound **2** as a colorless oil (4.5 g, 77% yield). TLC: R_f_ = 0.7 (PE/AcOEt = 5:1, UV_254_). ^1^H NMR (500 MHz, CDCl_3_) δ 10.61 (s, 1H), 9.81 (s, 1H), 7.10 (dd, *J* = 8.9, 3.0 Hz, 1H), 7.01 (d, *J* = 3.0 Hz, 1H), 6.87 (d, *J* = 8.9 Hz, 1H), 1.29 − 1.19 (m, 3H), 1.10 (d, *J* = 7.2 Hz, 18H). ^13^C NMR (126 MHz, CDCl_3_) δ 196.1, 156.0, 148.8, 129.7, 122.3, 120.4, 118.4, 17.9, 12.6. LRMS-ESI (*m*/*z*): calcd for C_16_H_27_O_3_Si [M+H]^+^ 295.2, found 295.6.

#### 3.2.2. Synthesis of 3-Bromo-2-Hydroxy-5-((Triisopropylsilyl)Oxy)Benzaldehyde (**3**)

In a 50 mL flask containing compound **2** (6.0 mmol, 1.75 g, 1.0 equiv.) and anhydrous pyridine (12 mmol, 0.96 mL, 2.0 equiv.) dissolved in DCM (15 mL), bromine (12 mmol, 0.61 mL, 2.0 equiv.) dissolved in DCM was added dropwise at room temperature (25 °C), and the reaction was stirred at this temperature for 3 h. After completion of the reaction, a saturated Na_2_SO_3_ solution was added to remove excess bromine, followed by the addition of DCM (15 mL), and the mixture was washed with water (10 mL × 3). The organic layer was dried over anhydrous Na_2_SO_4_, removed, and the residue was purified with silica gel chromatography (eluent: PE/AcOEt = 50/1) to afford the target compound **3** as a colorless gel (1.63 g, 73% yield). TLC: R_f_ = 0.72 (PE/AcOEt = 5:1, UV_254_). ^1^H NMR (500 MHz, CDCl_3_) δ 11.13 (s, 1H), 9.77 (s, 1H), 7.37 (d, *J* = 2.9 Hz, 1H), 7.01 (d, *J* = 2.9 Hz, 1H), 1.25 (ddt, *J* = 13.9, 9.9, 6.6 Hz, 3H), 1.11 (d, *J* = 7.2 Hz, 18H). ^13^C NMR (126 MHz, CDCl_3_) δ 195.6, 152.6, 149.1, 132.4, 122.1, 120.6, 111.0, 17.8, 12.5. LRMS-ESI (*m*/*z*): calcd for C_16_H_26_BrO_3_Si [M+H]^+^ 373.1, 375.1, found 373.1, 375.1.

#### 3.2.3. Synthesis of 3-Bromo-5-Hydroxy-2-Methoxybenzaldehyde (**4**)

In a 50 mL flask containing compound **3** (8.94 mmol, 3.3 g, 1,0 equiv.) and K_2_CO_3_ (17.88 mmol, 2.47 g, 2.0 equiv.) dissolved in anhydrous DMF (8 mL, stored with 4 Ǻ MS under N_2_), CH_3_I (17.88 mmol, 1.11 mL, 2.0 equiv.) was injected via syringe and the reaction was stirred at 10 °C for 4 h. After completion of the reaction, cooled AcOEt (30 mL) and water (5 mL) were added consecutively at this temperature. Then the organic layer was washed with water (10 mL × 5), dried over anhydrous Na_2_SO_4_, and removed. The crude residue was dissolved in anhydrous THF (10 mL), TBAF (8.94 mmol, 8.94 mL, 1.0 equiv.) was added, and the reaction was stirred at room temperature for 0.5 h. THF was removed under reduced pressure, and AcOEt (30 mL) was added. The organic layer was washed with water (10 mL × 3), dried over Na_2_SO_4_, and removed. The residue was purified with silica gel chromatography (eluent: PE/AcOEt = 20/1 − 5/1) to afford the target compound **4** [16] as a white powder (1.75 g, 85% yield). TLC: R_f_ = 0.3 (PE/AcOEt = 4:1, UV_254_). ^1^H NMR (500 MHz, CDCl_3_) δ 10.29 (s, 1H), 7.38 (d, *J* = 3.0 Hz, 1H), 7.28 (d, *J* = 3.2 Hz, 1H), 5.53 (s, 1H), 3.94 (s, 3H). ^13^C NMR (126 MHz, (CD_3_)_2_SO) δ 175.2, 140.4, 138.9, 116.9, 112.8, 104.6, 99.1, 49.8. LRMS-ESI (*m*/*z*): calcd for C_8_H_6_BrO_3_ [M − H]^−^ 229.0, 231.0, found 229.0, 231.0.

#### 3.2.4. Synthesis of 5-(3-(Benzyloxy)-4-Formylphenoxy)-3-Bromo-2-Methoxybenzaldehyde (**6**)

In a 25 mL flask containing compound **4** (1.95 mmol, 448 mg, 1.0 equiv.), 2-(benzyloxy)-4-fluorobenzaldehyde (**5**, 3.51 mmol, 807.3 mg, 1.8 equiv.), and K_2_CO_3_ (5.85 mmol, 806.8 mg, 3.0 equiv.) dissolved in anhydrous DMF (10 mL), the reaction mixture was heated at 100 °C overnight (about 18 h). After completion of the reaction, DMF was removed under reduced pressure, and AcOEt (30 mL) was added. The organic layer was washed with water (10 mL × 3), dried over Na_2_SO_4_, and removed, and the residue was purified with silica gel chromatography (eluent: PE/AcOEt = 15/1 − 2/1) to afford the target compound **6** as a colorless gel (0.8 g, 93% yield). TLC: R_f_ = 0.33 (PE/AcOEt = 4:1, UV_254_). ^1^H NMR (500 MHz, CDCl_3_) δ 10.44 (s, 1H), 10.32 (s, 1H), 7.85 (d, *J* = 8.7 Hz, 1H), 7.53 (d, *J* = 3.0 Hz, 1H), 7.45 (d, *J* = 3.0 Hz, 1H), 7.38 − 7.40 (m, 4H), 7.35 (ddd, *J* = 8.4, 5.4, 3.3 Hz, 1H), 6.62 (d, *J* = 2.2 Hz, 1H), 6.55 (dd, *J* = 8.7, 2.8 Hz, 1H), 5.13 (s, 2H), 4.01 (s, 3H). ^13^C NMR (126 MHz, CDCl_3_) δ 188.2, 188.2, 163.1, 162.8, 157.0, 152.1, 135.5, 131.3, 131.0, 130.7, 128.8, 128.5, 127.3, 121.3, 119.1, 118.3, 110.1, 102.9, 70.8, 63.8. LRMS-ESI (*m*/*z*): calcd for C_22_H_18_BrO_5_ [M+H]^+^ 441.0, 443.0, found 441.6, 443.5.

#### 3.2.5. Synthesis of 5-(3-(Benzyloxy)-4-Formylphenoxy)-4′-(Tert-Butoxy)-2-Methoxy-[1,1′-Biphenyl]-3-Carbaldehyde (**8**)

In a 15 mL Schlenk tube containing compound **6** (0.682 mmol, 300 mg, 1.0 equiv.), 4-*tert*-butyl-benzoboronic acid (**7**, 1.36 mmol, 265 mg, 2.0 equiv.), and K_2_CO_3_ (2.04 mmol, 282 mg, 3.0 equiv.) dissolved in anhydrous DMF (4 mL) and water (0.5 mL), Pd(OAc)_2_ (0.136 mmol, 30 mg, 0.2 equiv.) and PPh_3_ (0.34 mmol, 90 mg, 0.5 equiv.) were added and the solution was bubbled with N_2_ for 10 min, the mixture was heated at 80 °C in a nitrogen atmosphere overnight. After completion of the reaction, DMF was removed under reduced pressure, and AcOEt (20 mL) was added. The organic layer was washed with water (5 mL × 3), dried over Na_2_SO_4_, and removed. The residue was purified with silica gel chromatography (eluent: PE/AcOEt = 50/1 − 10/1) to afford the target compound **8** as a colorless gel (0.3 g, 86% yield). TLC: R_f_ = 0.3 (PE/DCM = 4:1, then DCM, UV_254_). ^1^H NMR (500 MHz, (CD_3_)_2_SO) δ 10.32 (s, 1H), 10.29 (s, 1H), 7.73 (d, *J* = 8.6 Hz, 1H), 7.54 (d, *J* = 8.6 Hz, 2H), 7.48 − 7.43 (m, 3H), 7.35 (t, *J* = 7.2 Hz, 2H), 7.33 − 7.30 (m, 2H), 7.09 (d, *J* = 8.6 Hz, 2H), 6.96 (d, *J* = 2.2 Hz, 1H), 6.65 (t, *J* = 9.3 Hz, 1H), 5.26 (s, 2H), 3.52 (s, 3H), 1.34 (s, 9H). ^13^C NMR (126 MHz, (CD_3_)_2_SO) δ 189.5, 187.7, 163.4, 162.4, 157.0, 155.3, 151.1, 137.4, 136.2, 130.7, 130.3, 130.1, 129.5, 128.5, 128.2, 128.1, 127.6, 125.0, 123.4, 120.5, 116.9, 115.1, 109.9, 103.7, 78.4, 70.2, 62.6, 28.6. LRMS-ESI (*m*/*z*): calcd for C_32_H_31_O_6_ [M+H]^+^ 511.2, found 511.5.

#### 3.2.6. Synthesis of 7-(Benzyloxy)-3-(4-(Tert-Butoxy)Phenyl)-2-Methoxydibenzo[b,d]Furan-1,8-Dicarbaldehyde (**8a**)

In a 10 mL vial containing compound **8** (0.12 mmol, 60 mg, 1.0 equiv.), Pd(OAc)_2_ (0.036 mmol, 8 mg, 0.3 equiv.), and AgOAc (0.24 mmol, 40 mg, 2.0 equiv.), PivOH (0.3 mL) was added, and the reaction mixture was heated at 130 °C for 24 h. PivOH was removed under reduced pressure, and AcOEt (10 mL) was added. The organic layer was washed with water (3 mL × 3), dried over Na_2_SO_4_, and removed, and the residue was purified with silica gel chromatography (eluent: PE/DCM = 10/1 − 1/1.5) to afford the target compound **8a** as a white solid (7.8 mg, 13% yield). TLC: R_f_ = 0.3 (PE/DCM = 4:1, then PE/DCM = 1:1, UV_254_). ^1^H NMR (700 MHz, CDCl_3_) δ 10.77 (s, 1H), 10.62 (s, 1H), 9.53 (s, 1H), 7.77 (s, 1H), 7.59 − 7.54 (m, 2H), 7.51 (d, *J* = 7.1 Hz, 2H), 7.44 (t, *J* = 7.5 Hz, 2H), 7.40 − 7.35 (m, 1H), 7.18 (s, 1H), 7.13 − 7.10 (m, 2H), 5.31 (s, 2H), 3.54 (s, 3H), 1.43 (s, 9H). ^13^C NMR (176 MHz, CDCl_3_) δ 190.9, 189.1, 162.8, 161.9, 158.9, 155.6, 153.5, 135.6, 134.1, 131.5, 129.7, 129.0, 128.8, 128.4, 127.3, 124.9, 124.1, 122.0, 118.9, 117.4, 95.7, 78.9, 70.9, 63.0, 28.9. LRMS-ESI (*m*/*z*): calcd for C_32_H_29_O_6_ [M+H]^+^ 509.2, found 509.4. The crystallization data for this compound were stored in the Cambridge Structural Database (CSD). CCDC 2475075 contains the supplementary crystallographic data for this paper. These data can be obtained free of charge from The Cambridge Crystallographic Data Centre via www.ccdc.cam.ac.uk/data_request/cif, accessed on 20 September 2020.

#### 3.2.7. Synthesis of 2-(Benzyloxy)-4-((4′-(Tert-Butoxy)-6-Methoxy-5-(Pivaloyloxy)-[1,1′-Biphenyl]-3-Yl)Oxy)Phenyl Pivalate (**9**)

In a 25 mL flask containing compound **8** (0.12 mmol, 60 mg, 1.0 equiv.) dissolved in anhydrous DCM (8 mL), *m*-CPBA (0.48 mmol, 95 mg (85 wt%), 4.0 equiv.) was added in portions at 0 °C, and then the reaction mixture was stirred at room temperature (25 °C) overnight. After the reaction was completed, a saturated Na_2_S_2_O_3_ solution was added to quench excess *m*-CPBA. Additional DCM (10 mL) was added, and the organic layer was washed with water (3 mL × 3), 10% NaOH solution (3 mL × 3), and removed. The residue was dissolved in MeOH (5 mL), and 2 M NaOH (2 mL, 10 equiv.) was added. The reaction was stirred at room temperature for about 1 h. After the reaction was completed, MeOH was removed, and AcOEt (15 mL) was added. The organic layer was washed with water (3 mL × 3), dried over Na_2_SO_4_, and removed. The residue was dried in vacuum overnight. The above crude mixture (0.062 mmol, 30 mg, 1.0 equiv.) dissolved in dry DCM (6 mL) was cooled to 0 °C, and Et_3_N (0.3 mmol, 42 μL, 5.0 equiv.) was added, followed by adding PivCl (0.28 mmol, 30 μL, 4.0 equiv.) dropwise and DMAP (0.006 mmol, 1.0 mg, 0.1 equiv.). The reaction mixture was further stirred at this temperature for about 3 h. After the reaction was completed, DCM (15 mL) and water (3 mL) were added, and the organic layer was washed with water (3 mL × 3), dried, and removed. The residue was purified with silica gel chromatography (eluent: PE/AcOEt = 50/1 − 40/1) to afford the target compound **9** as a colorless gel (30.1 mg, 38% yield over three steps from **8**). TLC: R_f_ = 0.6 (PE/DCM = 2:1, UV_254_). ^1^H NMR (500 MHz, CDCl_3_) δ 7.44 (d, *J* = 8.6 Hz, 2H), 7.37 (d, *J* = 6.4 Hz, 2H), 7.33 (t, *J* = 7.1 Hz, 2H), 7.30 (d, *J* = 6.9 Hz, 1H), 7.02 (d, *J* = 8.6 Hz, 2H), 6.97 (d, *J* = 8.6 Hz, 1H), 6.88 (d, *J* = 2.9 Hz, 1H), 6.76 (d, *J* = 2.7 Hz, 1H), 6.68 (d, *J* = 2.9 Hz, 1H), 6.62 (dd, *J* = 8.7, 2.6 Hz, 1H), 4.99 (s, 2H), 3.36 (s, 3H), 1.38 (s, 9H), 1.38 (s, 9H), 1.26 (s, 9H). ^13^C NMR (126 MHz, CDCl_3_) δ 176.8, 176.6, 167.3, 155.3, 155.2, 152.8, 151.2, 145.2, 136.6, 136.2, 132.0, 129.6, 128.4, 128.1, 127.8, 123.9, 123.2, 117.9, 112.7, 110.6, 105.2, 78.7, 70.9, 60.7, 39.2, 39.0, 29.0, 27.2, 27.2. LRMS-ESI (*m*/*z*): calcd for C_40_H_47_O_8_ [M+H]^+^ 655.3, found 655.3.

#### 3.2.8. Synthesis of 7-(Benzyloxy)-3-(4-(Tert-Butoxy)Phenyl)-2-Methoxydibenzo[b,d]Furan-1,8-Diyl Bis(2,2-Dimethylpropanoate) (**9a**)

In a 10 mL vial containing compound **9** (0.035 mmol, 23 mg), Pd(TFA)_2_ (3.5 mg, 0.01 mmol, 0.3 equiv.), and AgOAc (11.6 mg, 0.07 mmol, 2.0 equiv.), PivOH (0.3 mL) was added, and the mixture was heated at 130 °C for 10 h. PivOH was removed under reduced pressure, and AcOEt (6 mL) was added. The organic layer was washed with water (3 mL × 3), 10% NaOH solution (1 mL × 3), dried over Na_2_SO_4_, and removed. The residue was purified with silica gel chromatography (eluent: PE/DCM = 10/1 − 1/1) to afford the target compound **9a** as a colorless gel in 53% isolated yield (12 mg) with 13% (3 mg) of **9** recovered. TLC: R_f_ = 0.5 (PE/DCM = 2:1, UV_254_). ^1^H NMR (700 MHz, (CD_3_)_2_SO) δ 7.70 (s, 1H), 7.63 (s, 1H), 7.56 (d, *J* = 8.6 Hz, 2H), 7.44 (d, *J* = 6.9 Hz, 2H), 7.41 (t, *J* = 7.6 Hz, 2H), 7.35 (t, *J* = 7.2 Hz, 1H), 7.32 (s, 1H), 7.09 (d, *J* = 8.6 Hz, 2H), 5.22 (s, 2H), 3.36 (s, 3H), 1.46 (s, 9H), 1.36 (s, 9H), 1.23 (s, 9H). ^13^C NMR (126 MHz, (CD_3_)_2_SO) δ 175.9, 175.5, 154.9, 154.5, 152.3, 150.7, 145.0, 137.5, 136.6, 136.1, 133.4, 131.5, 129.7, 128.4, 128.2, 127.9, 123.3, 116.9, 114.2, 113.6, 110.2, 98.2, 78.2, 70.6, 60.8, 38.4, 28.6, 26.8, 26.7. LRMS-ESI (*m*/*z*): calcd for C_40_H_44_O_8_Na [M+Na]^+^ 675.3, found 675.3.

#### 3.2.9. Synthesis of Compound 2-(Benzyloxy)-4-((4′-(Tert-Butoxy)-5-((Dimethylcarbamoyl)Oxy)-6-Methoxy-[1,1′-Biphenyl]-3-Yl)Oxy)Phenyl Dimethylcarbamate (**10**)

Following a similar procedure for the preparation of compound **9**, replacing PivCl by (CH_3_)_2_NCOCl (5.0 equiv.), and using compound **8** (0.11 mmol, 58.7 mg, 1.0 equiv.), compound **10** (eluent: PE/AcOEt = 10/1 − 5:1) was synthesized as a colorless gel (45 mg, 62% yield over three steps from **8**). TLC: R_f_ = 0.4 (PE/AcOEt = 3:1, UV_254_). ^1^H NMR (500 MHz, CDCl_3_) δ 7.44 (d, *J* = 8.6 Hz, 2H), 7.39 (d, *J* = 6.7 Hz, 2H), 7.34 (t, *J* = 7.3 Hz, 2H), 7.31 − 7.27 (m, 1H), 7.06 (d, *J* = 8.7 Hz, 1H), 7.01 (d, *J* = 8.6 Hz, 2H), 6.86 (d, *J* = 3.0 Hz, 1H), 6.76 (d, *J* = 2.7 Hz, 1H), 6.75 (d, *J* = 3.0 Hz, 1H), 6.63 (dd, *J* = 8.6, 2.7 Hz, 1H), 5.03 (s, 2H), 3.42 (s, 3H), 3.13 (s, 3H), 3.05 (s, 3H), 3.02 (s, 3H), 2.97 (s, 3H), 1.38 (s, 9H). ^13^C NMR (126 MHz, CDCl_3_) δ 155.1, 154.9, 154.7, 154.4, 152.9, 151.5, 145.5, 145.4, 137.1, 136.6, 136.3, 132.2, 129.6, 128.4, 127.8, 127.2, 123.7, 123.6, 117.3, 112.8, 111.0, 105.8, 78.6, 70.7, 60.7, 36.9, 36.7, 36.5, 36.5, 28.9. LRMS-ESI (*m*/*z*): calcd for C_36_H_41_N_2_O_8_ [M+H]^+^ 629.3, found 629.3.

#### 3.2.10. Synthesis of 7-(Benzyloxy)-3-(4-(Tert-Butoxy)Phenyl)-2-Methoxydibenzo[b,d]Furan-1,8-Diyl Bis(Dimethylcarbamate) (**10a**)

In a 10 mL vial containing compound **10** (0.14 mmol, 86.1 mg), Pd(OAc)_2_ (0.04 mmol, 9.2 mg, 0.3 equiv.), and AgOAc (45.7 mg, 0.27 mmol, 2.0 equiv.), PivOH (0.3 mL) was added, and the mixture was heated at 130 °C for 10 h. PivOH was removed under reduced pressure, and AcOEt was added. The organic layer was washed with water, dried over Na_2_SO_4_, and removed, and the residue was purified with silica gel chromatography (eluent: PE/DCM = 5/1 − 2:1) to afford the target compound **10a** as a colorless gel (41.5 mg, 49% yield). TLC: R_f_ = 0.3 (PE/AcOEt = 3:1, UV_254_). ^1^H NMR (500 MHz, CDCl_3_) δ 7.56 − 7.51 (m, 3H), 7.44 (d, *J* = 6.9 Hz, 2H), 7.39 (t, *J* = 7.3 Hz, 2H), 7.35 (s, 1H), 7.33 (t, *J* = 7.2 Hz, 1H), 7.17 (s, 1H), 7.05 (d, *J* = 8.6 Hz, 2H), 5.16 (s, 2H), 3.45 (s, 3H), 3.32 (s, 3H), 3.13 (s, 3H), 3.08 (s, 3H), 2.99 (s, 3H), 1.40 (s, 9H). ^13^C NMR (126 MHz, CDCl_3_) δ 155.2, 154.9, 154.8, 154.1, 153.1, 150.8, 145.8, 138.3, 137.8, 136.5, 133.6, 132.9, 129.8, 128.5, 128.0, 127.3, 123.8, 118.1, 115.8, 115.2, 109.8, 97.4, 78.6, 71.0, 61.0, 37.1, 36.9, 36.8, 36.6, 28.9. LRMS-ESI (*m*/*z*): calcd for C_36_H_39_N_2_O_8_ [M+H]^+^ 627.3, found 627.3.

#### 3.2.11. Synthesis of 3′,4′-Bis(Benzyloxy)-5-(3-(Benzyloxy)-4-Formylphenoxy)-2-Methoxy-[1,1′-Biphenyl]-3-Carbaldehyde (**11**)

Following a similar procedure for the preparation of compound **8**, compound **6** (1.33 mmol, 587.6 mg, 1.0 equiv.) and (3,4-bis(benzyloxy)phenyl)boronic acid (1.6 mmol, 533 mg, 1.2 equiv.) were used to afford compound **11** (eluent: PE/AcOEt = 30/1 − 10:1) as a white powder (588 mg, 68%). TLC: R_f_ = 0.5 (PE/AcOEt = 5:1, UV_254_). ^1^H NMR (500 MHz, CDCl_3_) δ 10.42 (s, 1H), 10.41 (s, 1H), 7.83 (d, *J* = 8.6 Hz, 1H), 7.48 (t, *J* = 6.6 Hz, 3H), 7.45 (s, 1H), 7.44 (d, *J* = 3.0 Hz, 1H), 7.41 − 7.27 (m, 12H), 7.25 (d, *J* = 2.2 Hz, 1H), 7.23 (d, *J* = 3.0 Hz, 1H), 7.11 − 6.97 (m, 2H), 6.64 (d, *J* = 2.2 Hz, 1H), 6.55 (dd, *J* = 8.6, 2.4 Hz, 1H), 5.23 (s, 2H), 5.22 (s, 2H), 5.12 (s, 2H), 3.41 (s, 3H). ^13^C NMR (126 MHz, CDCl_3_) δ 189.4, 188.2, 163.9, 162.8, 157.6, 151.3, 149.2, 148.6, 137.5, 137.0, 137.0, 135.6, 130.9, 130.6, 129.1, 128.8, 128.7, 128.6, 128.5, 128.4, 127.9, 127.9, 127.4, 127.3, 127.3, 122.0, 120.9, 117.5, 115.8, 114.7, 109.7, 102.5, 71.3, 71.2, 70.7, 62.5. LRMS-ESI (*m*/*z*): calcd for C_42_H_35_O_7_ [M+H]^+^ 651.2, found 651.4.

#### 3.2.12. Synthesis of 2-(Benzyloxy)-4-((3′,4′-Bis(Benzyloxy)-6-Methoxy-5-(Pivaloyloxy)-[1,1′-Biphenyl]-3-Yl)Oxy)Phenyl Pivalate (**12**)

Following similar procedure for the preparation of compound **9**, compound **11** (0.32 mmol, 210 mg) was used to afford compound **12** (eluent: PE/AcOEt = 30/1 − 20/1) as a colorless gel (156 mg, 61% yield over three steps from **8**). TLC: R_f_ = 0.75 (PE/AcOEt = 5:1, UV_254_). ^1^H NMR (500 MHz, CDCl_3_) δ 7.47 (t, *J* = 6.6 Hz, 4H), 7.41 − 7.27 (m, 11H), 7.24 (d, *J* = 2.2 Hz, 1H), 7.04 (dd, *J* = 8.3, 2.1 Hz, 1H), 6.98 (s, 1H), 6.96 (s, 1H), 6.83 (d, *J* = 3.0 Hz, 1H), 6.75 (d, *J* = 2.7 Hz, 1H), 6.66 (d, *J* = 2.9 Hz, 1H), 6.60 (dd, *J* = 8.7, 2.7 Hz, 1H), 5.20 (s, 2H), 5.19 (s, 2H), 4.98 (s, 2H), 3.27 (s, 3H), 1.39 (s, 9H), 1.26 (s, 9H). ^13^C NMR (126 MHz, CDCl_3_) δ 176.7, 176.6, 155.2, 152.7, 151.2, 148.8, 148.5, 145.2, 145.1, 137.2, 137.2, 136.3, 136.2, 136.1, 130.5, 128.5, 128.4, 128.4, 128.1, 127.8, 127.8, 127.7, 127.5, 127.3, 123.2, 122.1, 117.9, 116.0, 114.6, 112.5, 110.5, 105.1, 71.3, 71.2, 70.8, 60.5, 39.1, 38.9, 27.2, 27.2. LRMS-ESI (*m*/*z*): calcd for C_50_H_51_O_9_ [M+H]^+^ 795.3, found 795.5.

#### 3.2.13. Synthesis of 7-(Benzyloxy)-3-(3,4-Bis(Benzyloxy)Phenyl)-2-Methoxydibenzo[b,d]Furan-1,8-Diyl Bis(2,2-Dimethylpropanoate) (**12a**)

Following similar procedure for the preparation of compound **9a**, compound **12** (0.055 mmol, 44 mg) was used to afford compound **12a** (eluent: PE/DCM = 10/1 − 1/1.5) as a colorless gel (18 mg, 41%, 51% brsm). TLC: R_f_ = 0.4 (PE/DCM = 4:1, then PE/DCM = 1:1, UV_254_). ^1^H NMR (500 MHz, CD_2_Cl_2_) δ 7.49 (t, *J* = 6.6 Hz, 4H), 7.47 − 7.42 (m, 2H), 7.42 (s, 1H), 7.41 − 7.38 (m, 5H), 7.37 (s, 1H), 7.36 (d, *J* = 1.5 Hz, 2H), 7.35 − 7.30 (m, 3H), 7.24 (s, 1H), 7.18 (dd, *J* = 8.2, 2.2 Hz, 1H), 7.06 (d, *J* = 8.4 Hz, 1H), 5.19 (s, 4H), 5.15 (s, 2H), 3.31 (s, 3H), 1.52 (s, 9H), 1.29 (s, 9H). ^13^C NMR (126 MHz, CD_2_Cl_2_) δ 177.1, 176.6, 155.6, 153.6, 151.4, 149.3, 149.1, 145.9, 138.7, 137.9, 137.9, 137.7, 136.7, 134.3, 131.6, 129.1, 129.1, 129.0, 128.8, 128.5, 128.4, 128.4, 128.2, 128.1, 122.9, 118.1, 116.6, 115.6, 115.3, 114.9, 110.5, 97.9, 71.8, 71.8, 71.7, 61.4, 39.9, 39.4, 27.7, 27.4. LRMS-ESI (*m*/*z*): calcd for C_50_H_49_O_9_ [M+H]^+^ 793.3, 793.4.

#### 3.2.14. Synthesis of Peniterphenyl A

In a 10 mL vial, compound **9a** (0.031 mmol, 20 mg, 1.0 equiv.) was dissolved in DCM (0.5 mL), and TFA (0.1 mL) was added dropwise, and the mixture was stirred at room temperature for 1 h. After the reaction was completed, DCM was removed under reduced pressure. Additional DCM (1.0 mL) was added, and the solvent was removed under reduced pressure; this procedure was repeated twice. The residue was dissolved in MeOH/THF mixture (1.0 mL, *v*/*v* = 1/1), followed by the addition of 2 M NaOH (0.62 mmol, 0.3 mL, 20 equiv.). The mixture was heated at 50 °C for 1 h. MeOH and THF were removed and the residue was dissolved in AcOEt (10 mL). The organic layer was washed with water (3 mL × 3) and removed. The residue was dissolved in MeOH (1.5 mL), and 10% Pd/C (4 mg, 20 wt%) and HCO_2_NH_4_ (0.62 mmol, 40 mg, 20 equiv.) were added, and the reaction mixture was heated at 85 °C for 2 h. MeOH was removed, and the residue was redissolved in AcOEt (10 mL). The organic layer was washed with water (3 mL × 3), dried over Na_2_SO_4_, and removed. The residue was dissolved in MeOH and purified by semi-preparative HPLC (RP-C18, MeOH/H_2_O = 60/40, flow rate = 3.0 mL/min, λ = 254 nm) to afford peniterphenyl A (t_R_ = 16 min) as a colorless gel (6.0 mg, 59% yield over three steps from **9a**). Its ^1^H/^13^C NMR signals were carefully assigned and listed in Table S1. The NMR data (Table S1, Figures S1 and S2) and the HRMS result (Figure S5) for synthesized peniterphenyl A were in excellent agreement with the natural one, indicating our successful synthesis of this natural product. TLC: R_f_ = 0.2 (PE/AcOEt = 1:1.5, UV_254_). ^1^H NMR (700 MHz, (CD_3_)_2_SO) δ 9.64 (s, 1H), 9.54 (s, 1H), 9.29 (s, 1H), 9.03 (s, 1H), 7.43 (d, *J* = 8.6 Hz, 2H), 7.39 (s, 1H), 6.96 (s, 1H), 6.89 (s, 1H), 6.84 (d, *J* = 8.6 Hz, 2H), 3.33 (s, 3H). ^13^C NMR (176 MHz, (CD_3_)_2_SO) δ 157.1, 153.1, 150.0, 145.9, 144.8, 142.5, 140.4, 132.4, 130.4, 129.3, 115.6, 114.7, 113.0, 107.6, 102.8, 98.7, 60.7. HRMS-ESI (*m*/*z*): calcd for C_19_H_13_O_6_ [M − H]^–^ 337.0718, found 337.0726.

#### 3.2.15. Synthesis of Peniterphenyl E

In a 15 mL Schlenk tube under a nitrogen atmosphere, compound **12a** (0.038 mmol, 30 mg, 1.0 equiv.) was dissolved in dry THF (3 mL). The mixture was cooled to 0 °C, and LiAlH_4_ (0.38 mmol, 15.0 mg, 10 equiv.) was added in portions. The reaction was stirred at this temperature for 1 h. Upon completion, a saturated NH_4_Cl solution was added dropwise. AcOEt (10 mL) was then added, and the mixture was washed with saturated NH_4_Cl (2 mL × 2) solution and H_2_O (2 mL × 2). The organic layer was removed under reduced pressure, and the residue was subjected to short silica gel chromatography (eluent: PE/AcOEt = 10/1). The crude product was dissolved in MeOH (2 mL), followed by the addition of HCO_2_NH_4_ (1.52 mmol, 96 mg, 40 equiv.) and 10% Pd/C (6 mg, 20 wt%). The reaction mixture was heated at 85 °C for 1 h. After completion, the solution was directly purified by semi-preparative HPLC (RP-C18, MeOH/H_2_O = 50/50, flow rate = 3.0 mL/min, λ = 254 nm) to afford peniterphenyl E (t_R_ = 14 min) as a white gel (4.0 mg, 30% yield over two steps from **12a**). Its ^1^H/^13^C NMR signals were carefully assigned and listed in Table S2. The NMR data (Table S2, Figures S3 and S4) and the HRMS result (Figure S6) for synthesized peniterphenyl E were in excellent agreement with the natural one, indicating our successful synthesis of this natural product. TLC: R_f_ = 0.2 (PE/AcOEt = 1:3, UV_254_). ^1^H NMR (700 MHz, CD_3_OD) δ 7.47 (s, 1H), 7.12 (d, *J* = 2.2 Hz, 1H), 6.97 (dd, *J* = 8.2, 2.2 Hz, 1H), 6.93 (s, 1H), 6.85 (s, 1H), 6.83 (d, *J* = 8.2 Hz, 1H), 3.43 (s, 3H). ^13^C NMR (176 MHz, CD_3_OD) δ 154.9, 152.0, 146.5, 146.1, 145.9, 145.8, 143.0, 141.2, 133.9, 131.9, 121.8, 117.4, 116.6, 116.2, 113.8, 108.6, 103.7, 98.9, 61.0. HRMS-ESI (*m*/*z*): calcd for C_19_H_13_O_7_ [M − H]^–^ 353.0667, found 353.0672.

## 4. Conclusions

In summary, we accomplished the first total syntheses of peniterphenyls A and E in thirteen and twelve steps, respectively. This approach circumvented the need for Ullmann-type coupling and the preparation of unique benzoboronic acids. The synthesis of key intermediate **4** enabled three site-selective modifications around the dibenzofuran scaffold: C5′-*O*-methylation, C4′-arylation, and C6′-hydroxylation. Direct C(sp^2^)–H/C(sp^2^)–H oxidative coupling was achieved with high selectivity through the strategic use of the substituent directing effect of the arene ring and steric control. This synthetic route provides a practical and straightforward approach for the preparation of *p*-terphenyl analogs. With a sufficient quantity of peniterphenyl A, future studies will focus on elucidating its mechanism of action against HSV-1/2.

## 5. Patents

We declare an applied Chinese patent (CN202511114605.8) that is relevant to the work disclosed in this manuscript.

## Figures and Tables

**Table 1:** Optimization of cyclization conditions using pivalate or *N*,*N*-dimethylcarbamate as directing group.

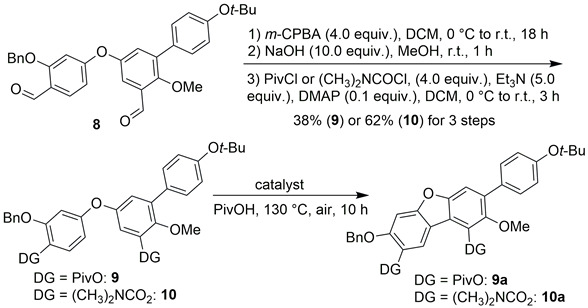			
**Entr** **y ^a^**	**DG**	**Catalyst**	**Yield (%) of** **9/** **9a or** **10/** **10a**
1 ^b^	PivO	Pd(OAc)_2_ (0.3 equiv.)/AgOAc (2.0 equiv.)	7/53
2 ^c^	PivO	Pd(OAc)_2_ (0.3 equiv.)/AgOAc (3.0 equiv.)	0/46
3	PivO	Pd(OAc)_2_ (0.3 equiv.)/AgOAc (6.0 equiv.)	0/32
4 ^d^	PivO	Pd(OAc)_2_ (0.3 equiv.)/AgOAc (6.0 equiv.)	0/32
5	PivO	Pd(OAc)_2_ (0.3 equiv.)	35/29
**6 ^e^**	**PivO**	**Pd(TFA)_2_ (0.3 equiv.)/AgOAc (2.0 equiv.)**	**22 (13)/60 (53)**
7	Me_2_NCO_2_	Pd(TFA)_2_ (0.3 equiv.)/AgOAc (2.0 equiv.)	4/60
8 ^e^	Me_2_NCO_2_	Pd(OAc)_2_ (0.3 equiv.)/AgOAc (2.0 equiv.)	2 (0)/54 (49)
9	Me_2_NCO_2_	Pd(TFA)_2_ (0.3 equiv.)/AgOAc (3.0 equiv.)	0/42

^a^ 0.02 mmol **9** or **10** was used in the reactions, the yields were determined by ^1^H NMR analysis of crude mixture using 1,3,5-triisopropylbenzene as an internal standard, and the yields of the cyclic site-isomer in all cases were <5% monitored by ^1^H NMR analysis. ^b^ The reaction time was 4 h. ^c^ The reaction was performed at 160 °C for 1 h. ^d^ The reaction was performed under N_2_ atmosphere for 1.5 h. ^e^ Isolated yield indicated in parentheses, and a 71% NMR or 60% isolated yield of **9a** based on recovery of the starting material.

## Data Availability

The data that support the findings of this study are available in the Supplementary Materials of this article.

## Supplementary Materials

The following supporting information can be downloaded at https://www.mdpi.com/article/10.3390/md23110437/s1. Figure S1. ^1^H NMR spectrum of peniterphenyl A. Figure S2. ^13^C NMR spectrum of peniterphenyl A. Figure S3. ^1^H NMR spectrum of peniterphenyl E. Figure S4. ^13^C NMR spectrum of peniterphenyl E. Figure S5. HRMS spectraof peniterphenyl A. Figure S6 HRMS spectra of peniterphenyl E. Figure S7. ^1^H NMR spectrum of compound **2**. Figure S8. ^13^C NMR spectrum of compound **2**. Figure S9. ^1^H NMR spectrum of compound **3.** Figure S10. ^13^C NMR spectrum of compound **3**. Figure S11. ^1^H NMR spectrum of compound **4**. Figure S12. ^13^C NMR spectrum of compound **4**. Figure S13. ^1^H NMR spectrum of compound **6**. Figure S14. ^13^C NMR spectrum of compound **6**. Figure S15. ^1^H NMR spectrum of compound **8**. Figure S16. ^13^C NMR spectrum of compound **8**. Figure S17. ^1^H NMR spectrum of compound **8a**. Figure S18. ^13^C NMR spectrum of compound **8a**. Figure S19. ^1^H NMR spectrum of compound **9**. Figure S20. ^13^C NMR spectrum of compound **9**. Figure S21. ^1^H NMR spectrum of compound **9a**. Figure S22. ^13^C NMR spectrum of compound **9a**. Figure S23. ^1^H NMR spectrum of compound **10**. Figure S24. ^13^C NMR spectrum of compound **10**. Figure S25. ^1^H NMR spectrum of compound **10a**. Figure S26. ^13^C NMR spectrum of compound **10a**. Figure S27. ^1^H NMR spectrum of compound **11**. Figure S28. ^13^C NMR spectrum of compound **11**. Figure S29. ^1^H NMR spectrum of compound **12**. Figure S30. ^13^C NMR spectrum of compound **12**. Figure S31. ^1^H NMR spectrum of compound **12a**. Figure S31. ^1^H NMR spectrum of compound **12a.** Figure S32. ^13^C NMR spectrum of compound **12a**. Table S1. ^1^H (700 MHz)/^13^C (176MHz) NMR (in DMSO-*d*6) signals comparison of natural and synthetic peniterphenyl A. Table S2. ^1^H (700 MHz)/^13^C (176MHz) NMR (in CD3OD) signals comparison of natural and synthetic peniterphenyl E. Table S3. Conditions screening for furan unit formation using aldehyde as the directing group. Table S4. Conditions screening of the furan formation step. Table S5. Crystal data and structure refinement for **8a**. Table S6. Fractional Atomic Coordinates (×104) and Equivalent Isotropic Displacement Parameters (Å^2^ × 103) for **8a**. U_eq_ is defined as 1/3 of of the trace of the orthogonalised U_IJ_ tensor. Table S7. Anisotropic Displacement Parameters (Å^2^ × 103) for **8a**. The Anisotropic displacement factor exponent takes the form: -2π^2^[h^2^a*^2^U_11_ + 2hka*b*U_12_+…]. Table S8. Bond Lengths for **8a**. Table S9. Bond Angles for **8a**. Table S10. Torsion Angles for **8a**. Table S11. Hydrogen Atom Coordinates (Å × 104) and Isotropic Displacement Parameters (Å^2^ × 103) for **8a**.

## Abbreviations

HSV 1/2	herpes simplex virus 1/2
PLA2	phospholipase A2
S_N_Ar	nucleophilic aromatic substitution
TFA	trifluoroacetic Acid
OMOM	methoxymethoxy
PivCl	pivaloyl chloride
IPSCl	triisopropylsilyl chloride
PivOH	pivalic acid
PivO	pivalate
(CH_3_)_2_NCO_2_	*N*,*N*-dimethylcarbamate
brsm	based on recovery of the starting material
BnO	benzyloxyl
HPLC	high-performance liquid chromatograph
NMR	nuclear magnetic resonance
DMF	*N*,*N*′-dimethylformamide
DCM	dichloromethane
DMAP	4-dimethylaminopyridine
PE	petroleum ether
AcOEt	ethyl estate
*m*-CPBA	3-chloroperbenzoic acid
MeOH	methanol
LRMS	low-resolution mass spectrometry
HRMS	high-resolution mass spectrometry
ESI	electrospray ionization
UV_254_	ultraviolet 254 nm
TLC	thin layer chromatography
R_f_	retention factor
