# Supplementary material for: Total Synthesis of Peniterphenyls A and E
Source: Mar Drugs. 2025 Nov 14;23(11):437. doi: 10.3390/md23110437 (PMC12654094; doi:10.3390/md23110437)

Surpporting Information

Total Synthesis of Peniterphenyls A and E

	TABLE OF CONTENTS	PAGE
1	Comparison of NMR signals for natural and synthetic peniterphenyls A and E	S2-3
2	NMR spectra of synthetic peniterphenyls A and E	S4-5
3	HRMS spectra of synthetic peniterphenyls A and E	S6
4	Conditions screening at furan formation step	S7-9
5	Crystal Experimental Analysis of **8a**	S10-18
6	Copies of NMR spectra of compounds	S19-31

1. Comparison of NMR signals for natural and synthetic peniterphenyls A and E

Table S1. ^{1}H (700 MHz)/^{13}C (176 MHz) NMR (in DMSO-d_6) signals comparison of natural and synthetic peniterphenyl A

	**Natural peniterphenyl A**[a]		**Synthetic peniterphenyl A**			
position	δC	δH	δC	δH	$\delta\Delta$C	$\delta\Delta$H
1	114.7, C		114.7, C		0	
2	150.0, C		150.0, C		0	
3	98.7, CH	6.97, s	98.7, CH	6.96, s	0	0.01
4	145.9, C		145.8, C		0.1	
5	142.5, C		142.5, C		0	
6	107.6, CH	7.40, s	107.6, CH	7.39, s	0	0.01
1’	113.0, C		112.9, C		0.1	
2’	153.1, C		153.1, C		0	
3’	102.8, CH	6.89, s	102.7, CH	6.89, s	0.1	0
4’	132.4, C		132.4, C		0	
5’	140.4, C		140.4, C		0	
6’	144.8, C		144.8, C		0	
1’’	129.3, C		129.2, C		0.1	
2’’	130.4, CH	7.43, d (8.5)	130.4, CH	7.43, d (8.6)	0	0
3’’	115.6, CH	6.85, d (8.5)	115.6, CH	6.84, d (8.4)	0	0.01
4’’	157.1, C		157.1, C		0	
5’’	115.6, CH	6.85, d (8.5)	115.6, CH	6.84, d (8.4)	0	0.01
6’’	130.4, CH	7.43, d (8.5)	130.4, CH	7.43, d (8.6)	0	0
5’-OMe	60.8, CH_3	3.34,s	60.7, CH_3	3.33, s	0.1	0.01

[a] Data copied from reference: *J. Nat. Prod. 2021, 84, 2822−2831* (where peniterphenyl A was also described as compound **1**)

Table S2. ^{1}H (700 MHz)/^{13}C (176 MHz) NMR (in CD_3OD) signals comparison of natural and synthetic peniterphenyl E

	**Natural peniterphenyl E**[a]		**Synthetic peniterphenyl E**			
position	δC	δH	δC	δH	$\delta\Delta$C	$\delta\Delta$H
1	116.6, C		116.6, C		0	
2	152.0, C		152.0, C		0	
3	98.9, CH	6.92, s	98.9, CH	6.93, s	0	-0.01
4	146.1, C		146.1, C		0	
5	142.9, C		143.0, C		-0.1	
6	108.6, CH	7.46, s	108.6, CH	7.47, s	0	-0.01
1’	113.8, C		113.8, C		0	
2’	154.9, C		154.9, C		0	
3’	103.7, CH	6.84, s	103.7, CH	6.85, s	0	-0.01
4’	133.9, C		133.9, C		0	
5’	141.2, C		141.2, C		0	
6’	146.5, C		146.5, C		0	
1’’	131.9, C		131.9, C		0	
2’’	117.4, CH	7.11, d (2.0)	117.4, CH	7.12, d (2.2)	0	-0.01
3’’	145.9, CH		145.9, CH		0	
4’’	145.7, C		145.8, C		-0.1	
5’’	116.2, CH	6.82, d (8.1)	116.2, CH	6.83, d (8.2)	0	-0.01
6’’	121.8, CH	6.96, dd (8.1，2.0)	121.8, CH	6.97, dd (8.2, 2.2)	0	
5’-OMe	61.0, CH_3	3.42, s	61.0, CH_3	3.43, s	0	-0.01

[a] Data copied from reference: *J. Nat. Prod.* ***1998,*** *61, 1115-1119* (where peniterphenyl E was also described as compound **2**)

2. NMR spectra of synthetic peniterphenyls A and E

Figure S1. ^{1}H NMR spectrum of peniterphenyl A

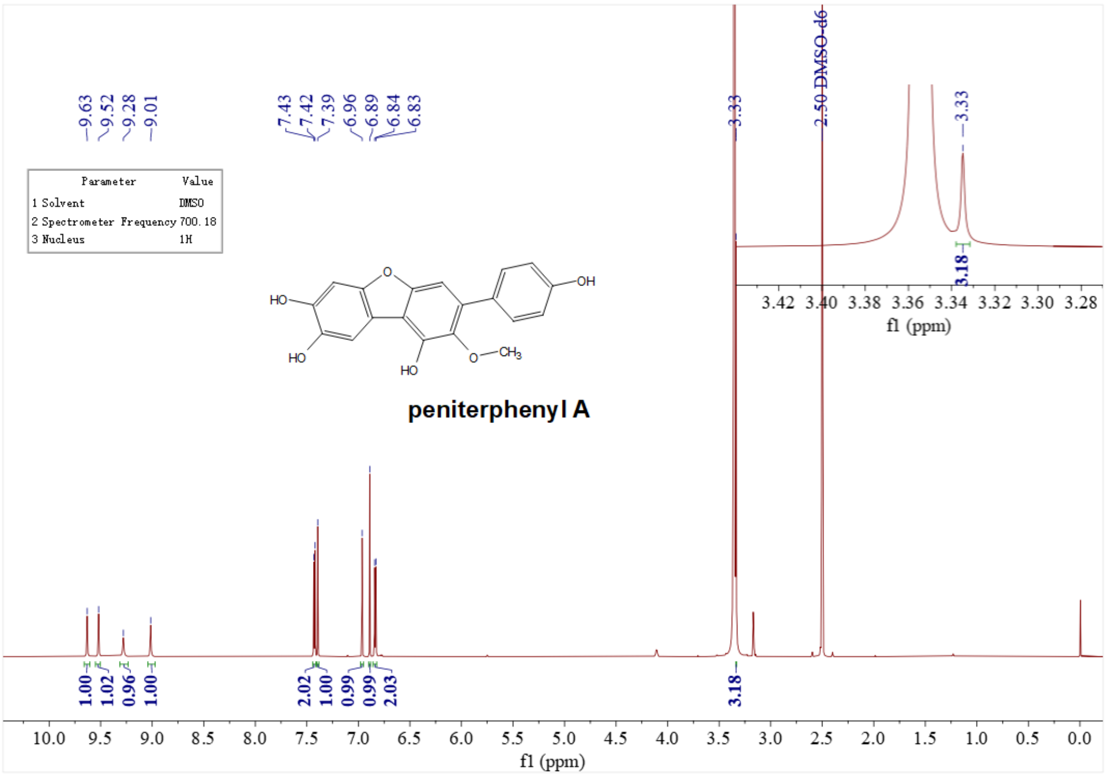

Figure S2. ^{13}C NMR spectrum of peniterphenyl A

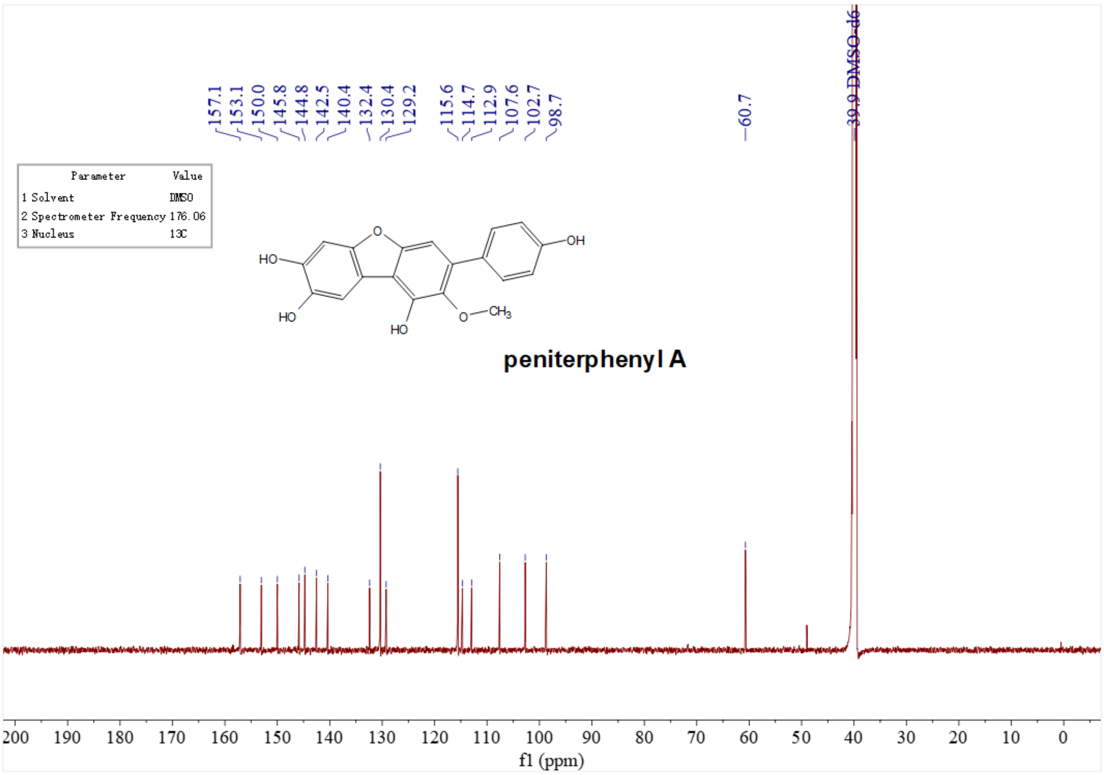

Figure S3. ^{1}H NMR spectrum of peniterphenyl E

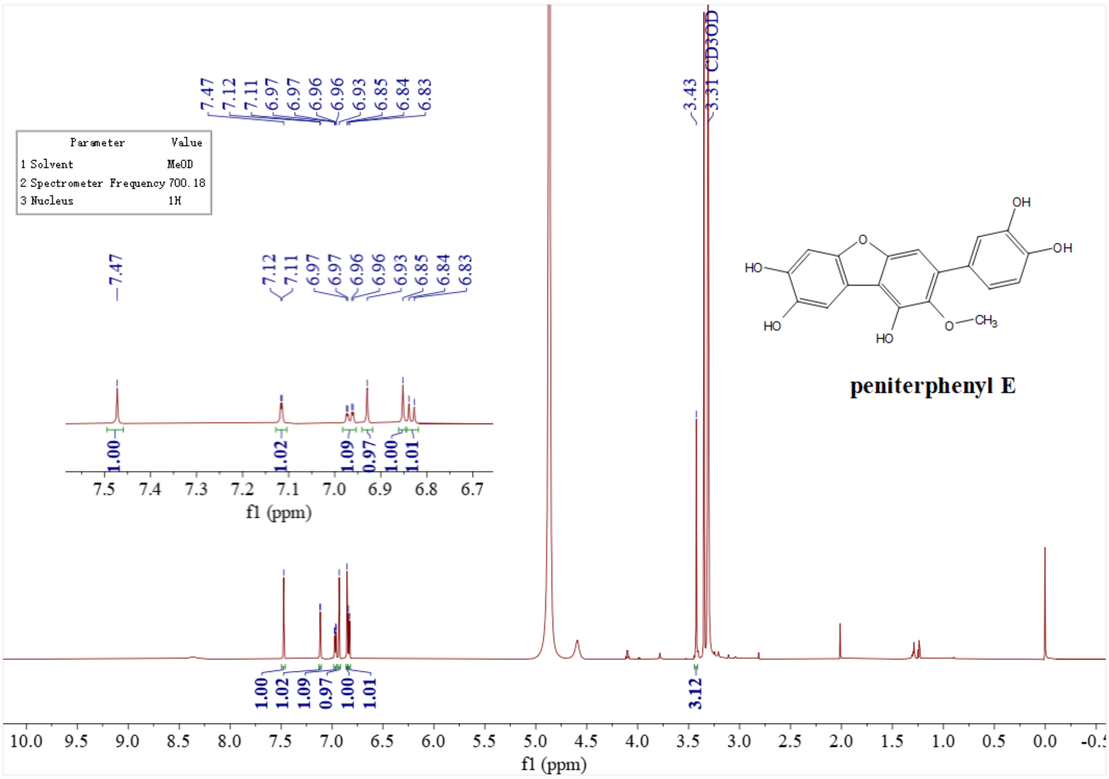

Figure S4. ^{13}C NMR spectrum of peniterphenyl E

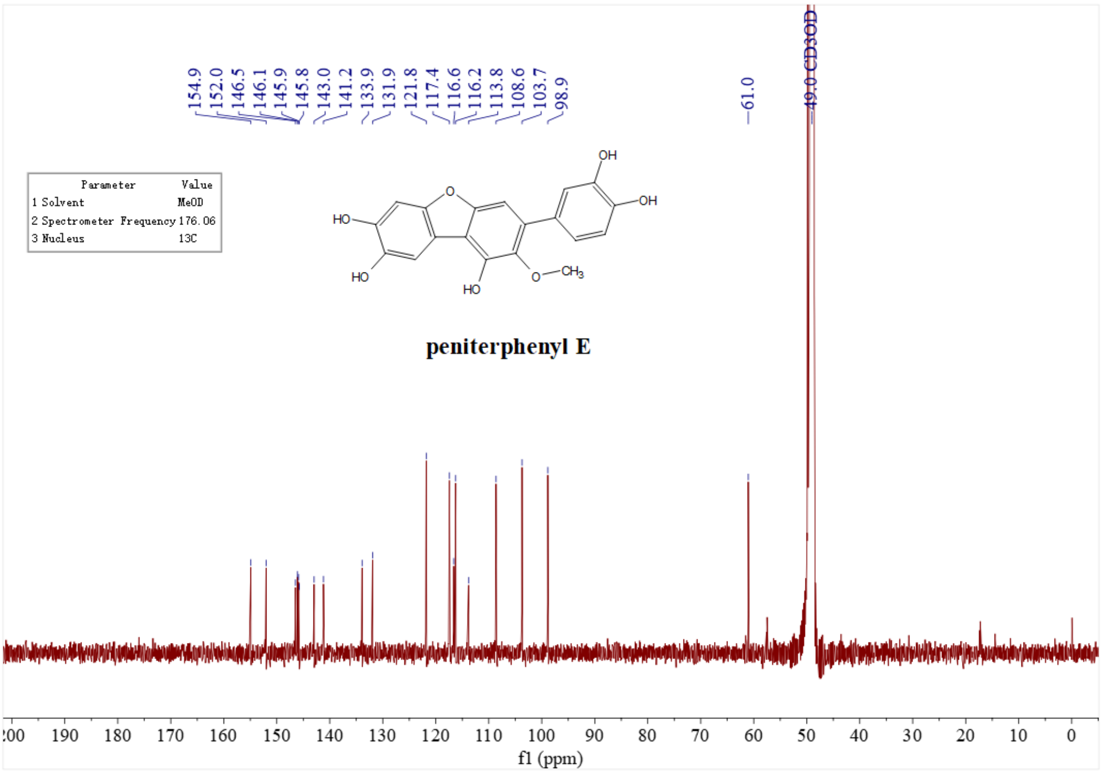

3. HRMS spectra of synthetic peniterphenyls A and E

Figure S5. HRMS spectra of peniterphenyl A

Mass Spectrum SmartFormula Report

Analysis Info

Analysis Name	D:\Data\MS\data\202504\liaoshengrong_LSR12-29-1_neg_23_01_19777.d	Acquisition Date	4/28/2025 2:52:19 PM	
Method	LC_Direct Infusion_neg_70-500mz.m	Operator	SCSIO	
Sample Name	liaoshengrong_LSR12-29-1_neg	Instrument	maXis	255552.00029
Comment				

Acquisition Parameter

Source Type	ESI	Ion Polarity	Negative	Set Nebulizer	0.4 Bar
Focus	Active	Set Capillary	4200 V	Set Dry Heater	180 °C
Scan Begin	100 m/z	Set End Plate Offset	-500 V	Set Dry Gas	4.0 l/min
Scan End	1500 m/z	Set Charging Voltage	0 V	Set Divert Valve	Waste
		Set Corona	0 nA	Set APCI Heater	0 °C

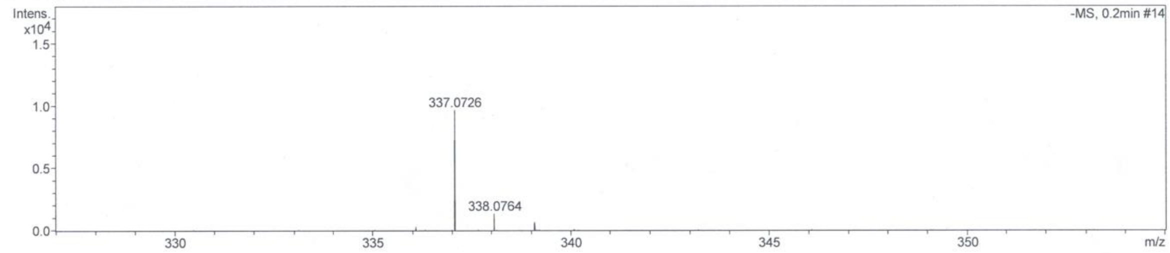

Meas. m/z	#	Ion Formula	Score	m/z	err [ppm]	err [mDa]	mSigma	rdb	e⁻ Conf	N-Rule
337.0726	1	C19H13O6	100.00	337.0718	2.6	0.9	38.4	13.5	even	ok
675.1518	1	C38H27O12	100.00	675.1508	-1.5	-1.0	220.2	25.5	even	ok

Figure S6 HRMS spectra of peniterphenyl E

Mass Spectrum SmartFormula Report

Analysis Info

Analysis Name	D:\Data\MS\data\202507\liaoshengrong_LSR12-44-21_neg_3_01_20421.d	Acquisition Date	7/28/2025 3:23:43 PM	
Method	LC_Direct Infusion_neg_70-500mz.m	Operator	SCSIO	
Sample Name	liaoshengrong_LSR12-44-21_neg	Instrument	maXis	255552.00029
Comment				

Acquisition Parameter

Source Type	ESI	Ion Polarity	Negative	Set Nebulizer	0.4 Bar
Focus	Active	Set Capillary	4200 V	Set Dry Heater	180 °C
Scan Begin	100 m/z	Set End Plate Offset	-500 V	Set Dry Gas	4.0 l/min
Scan End	1500 m/z	Set Charging Voltage	0 V	Set Divert Valve	Waste
		Set Corona	0 nA	Set APCI Heater	0 °C

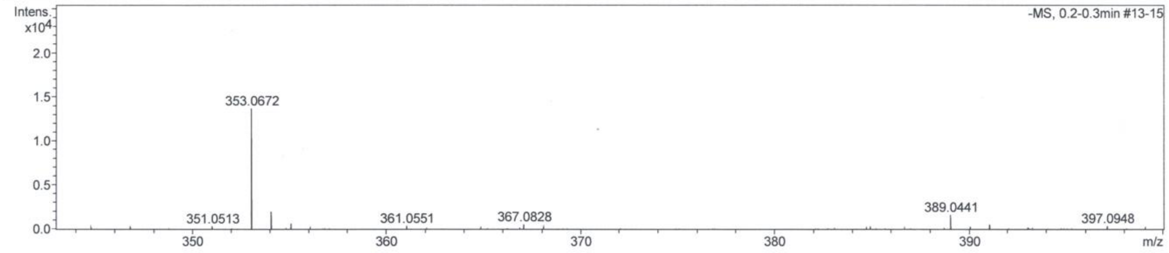

Meas. m/z	#	Ion Formula	Score	m/z	err [ppm]	err [mDa]	mSigma	rdb	e⁻ Conf	N-Rule
353.0672	1	C19H13O7	100.00	353.0667	1.6	0.6	32.4	13.5	even	ok
389.0441	1	C19H14ClO7	100.00	389.0434	1.9	0.7	159.1	12.5	even	ok

4. Conditions screening at furan formation step.

Table S3. Conditions screening for furan unit formation using aldehyde as the directing group

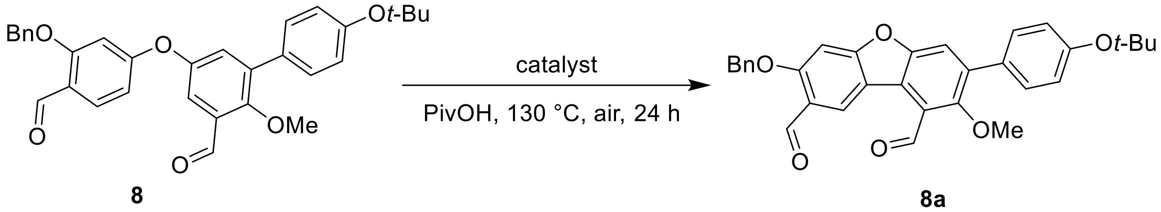

entry[a]	catalyst	yield of 8/8a
1[b]	$Pd(OAc)_2$ (0.3 equiv.)	100/0
2	$Pd(OAc)_2$ (0.3 equiv.)	29/16
3	$Pd(OAc)_2$ (0.3 equiv.)/K_2CO_3 (0.3 equiv.)	7/9
4	$Pd(OAc)_2$ (0.3 equiv.)/K_2CO_3 (0.3 equiv.)/$Cu(OAc)_2$ (3.0 equiv.)	37/10
5[c]	$Pd(OAc)_2$ (0.3 equiv.)/AgOAc (2.0 equiv.)	25/15 (13)
6[c]	$Pd(OAc)_2$ (0.3 equiv.)/AgOAc (6.0 equiv.)	27/10 (9)

[a] 0.02 mmol **8** was used, the yields were determined by ^{1}H NMR analysis of crude mixture using 1,3,5-triisopropylbenzene as an internal standard, and the yields of the cyclic site-isomers in all cases were < 5% monitored by ^{1}H NMR analysis.

[b] The reaction temperature was 25 ˚C.

[c] Isolated yield indicated in parentheses.

Table S4. Conditions screening of the furan formation step

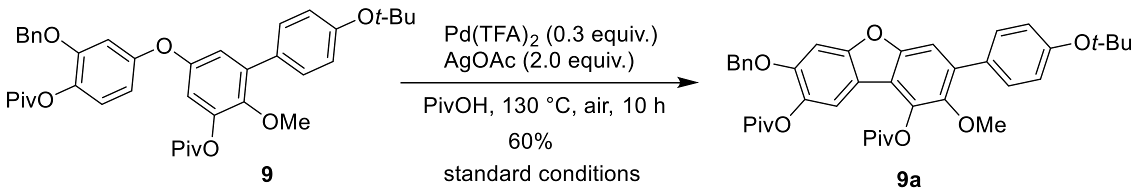

entry[a]	variations from the standard conditions	yield (%) of 9/9a
1	AcOH instead of PivOH	0/0
2	diglyme instead of PivOH	0/0
3	NMM instead of PivOH	88/0
4	DMSO instead of PivOH	49/0
5	Toluene instead of PivOH	19/3
6	DMF instead of PivOH	73/10
7	DCE instead of PivOH	9/19
8	using 3.0 equiv. AgOAc, in HFIP, 20 h	9/22
9	AgTFA instead of AgOAc	0/33
10	Ag_2CO_3 instead of AgOAc	0/33
11	$AgSbF_6$ instead of AgOAc	0/6
12	$AgNO_3$ instead of AgOAc	0/6
13	$AgBF_4$ instead of AgOAc	0/0
14	$AgNTf_2$ instead of AgOAc	0/0
15	AgOTf instead of AgOAc	0/0
16	$Pd(HFha)_2$ instead of $Pd(TFA)_2$	8/50
17	$Pd(OPiv)_2$ instead of $Pd(TFA)_2$	7/52
18	$Pd(COD)_2$ instead of $Pd(TFA)_2$	53/18
19	$Pd(acac)_2$ instead of $Pd(TFA)_2$	47/37
20	$Pd(PPh_3)_4$ instead of $Pd(TFA)_2$	20/40
21	$Pd(dppf)Cl_2$ instead of $Pd(TFA)_2$	81/0
22	$Pd(PPh_3)_2Cl_2$ instead of $Pd(TFA)_2$	86/0

23	using 2.5 equiv. AgOAc	5/48
24	using 3.0 equiv. AgOAc	0/44
25	using 0.4 equiv. $Pd(TFA)_2$	14/55
26	using 0.5 equiv. $Pd(TFA)_2$, 4 h	10/40
27	increasing reaction time to 24 h	9/52
28	0.3 equiv. K_2CO_3 instead of 2 equiv. AgOAc	24/24
29	no AgOAc, 100 ˚C	91/0
30	6.0 equiv. AgOAc, 100 ˚C	0/23
31	AgOAc (2.0 equiv.) added in portions (0.5 equiv. × 4)	17/52
32	$[RuCl_2(pcymene)]_2$ (0.1 equiv.)/K_2CO_3 (1.0 equiv.)/ $Cu(OAc)_2$ (2.0 equiv.), PivOH, 140 ˚C, N_2, 7 h	54/0
33	$[Cp^*Rh(MeCN)_3][SbF_6]_2$ (0.04 equiv.)/K_2CO_3 (1.0 equiv.)/ $Cu(OAc)_2$ (2.0 equiv.), PivOH (1.0 equiv.), DMF, 140 ˚C, N_2, 7 h	73/0

[a] 0.02 mmol **9** was used in the reactions, the yields were determined by ^{1}H NMR analysis of crude mixture using 1,3,5-triisopropylbenzene as an internal standard, and the yields of the cyclic site-isomers in all cases were < 5% monitored by ^{1}H NMR.

5. Crystal Experimental Analysis of 8a

A suitable crystal was selected on a Kappa single diffractometer (XtaLAB AFC12 (RINC)). The crystal was kept at 99.99(17) K during data collection. Using Olex2 [1], the structure was solved with the SHELXT [2] structure solution program via Intrinsic Phasing and refined with the SHELXL [2] refinement package via Least Squares minimisation.

1. Dolomanov, O.V., Bourhis, L.J., Gildea, R.J, Howard, J.A.K. & Puschmann, H. (2009), J. Appl. Cryst. 42, 339-341.
2. Sheldrick, G.M. (2015). Acta Cryst. A71, 3-8.

Compound 8a: CCDC 2475075

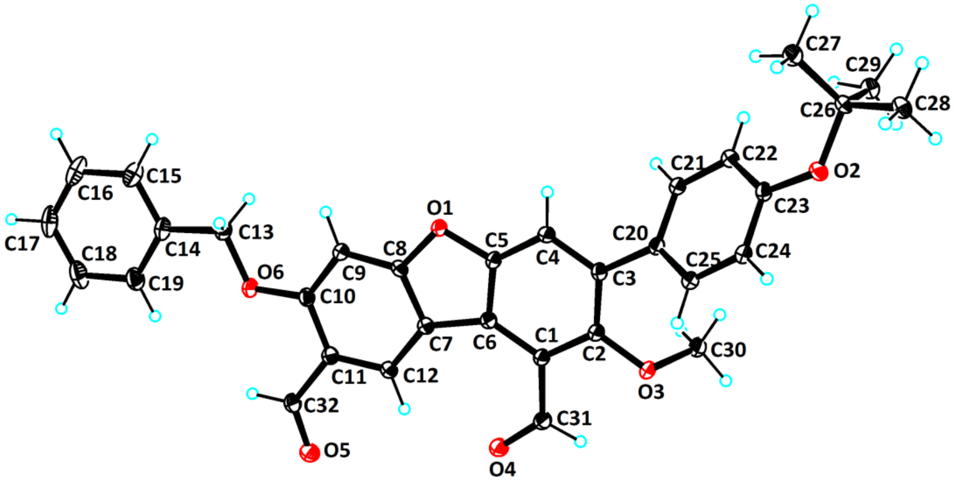

The crystallization data for this compound was stored in Cambridge Structural Database (CSD). It can be obtained freely at The Cambridge Crystallographic Data Centre (https://www.ccdc.cam.ac.uk).

Table S5. Crystal data and structure refinement for **8a.**

Identification code	**8a**
Empirical formula	$C_{22}H_{18}O_6$
Formula weight	378.36
Temperature/K	99.99(17)

Crystal system	monoclinic
Space group	$P2_1/n$
a/Å	11.8039(5)
b/Å	11.0382(3)
c/Å	14.3673(5)
α/°	90
β/°	107.430(4)
γ/°	90
Volume/$Å^3$	1786.02(12)
Z	4
ρ_{calc} g/cm^3	1.407
μ/mm^{-1}	0.854
F(000)	792.0
Crystal size/mm^3	0.23 × 0.13 × 0.04
Radiation	Cu Kα (λ = 1.54184)
2Θ range for data collection/°	8.54 to 148.34
Index ranges	$-14 \leq h \leq 13$, $-13 \leq k \leq 13$, $-16 \leq l \leq 17$
Reflections collected	8727
Independent reflections	3497 [R_{int} = 0.0435, R_{sigma} = 0.0511]
Data/restraints/parameters	3497/0/257
Goodness-of-fit on F^2	1.064
Final R indexes [I>=2σ (I)]	R_1 = 0.0555, wR_2 = 0.1505
Final R indexes [all data]	R_1 = 0.0734, wR_2 = 0.1597
Largest diff. peak/hole / $eÅ^{-3}$	0.92/-0.35

Table S6. Fractional Atomic Coordinates ($\times 10^4$) and Equivalent Isotropic Displacement Parameters ($Å^2 \times 10^3$) for **8a**. U_{eq} is defined as 1/3 of of the trace of the orthogonalised U_{IJ} tensor.

**Atom**	***x***	***y***	***z***	**U(eq)**
O9	780.7(12)	5123.9(11)	6446.8(10)	30.3(3)
O28	-1254.6(12)	8556.2(11)	4917.8(10)	30.7(3)
O7	-3521.8(13)	5077.1(11)	5106.4(10)	31.8(3)
O8	-3415.3(12)	7376.0(12)	4544.3(9)	29.3(3)
O17	3203.1(13)	10135.9(12)	6153.2(11)	35.5(4)
O24	8458.8(13)	6355.2(13)	8238.3(11)	36.6(4)
C4	-257.7(18)	6789.7(16)	5694.8(13)	27.9(4)
C2	-2373.2(17)	6758.1(16)	4996.7(13)	27.7(4)
C10	1569.7(18)	6049.7(16)	6496.1(14)	27.7(4)
C5	-337.5(18)	5591.1(17)	5967.2(14)	28.1(4)
C1	-2410.6(18)	5552.4(17)	5314.9(14)	27.9(4)
C15	2772.7(18)	5960.1(16)	6923.6(14)	29.9(4)
C11	985.7(18)	7108.3(16)	6040.6(13)	27.4(4)
C14	3474.5(18)	6976.5(17)	6911.2(14)	28.3(4)
C21	7250.2(18)	6482.7(17)	7976.8(15)	30.8(4)
C3	-1312.6(18)	7385.7(16)	5186.7(13)	27.1(4)
C13	2906.3(18)	8068.6(16)	6484.5(14)	28.3(4)
C18	4780.3(18)	6852.9(16)	7291.1(14)	28.9(4)
C20	6748.1(19)	7091.3(18)	7099.6(15)	32.7(4)
C12	1672.9(18)	8128.3(16)	6047.9(13)	28.3(4)
C6	-1373.7(18)	4934.7(17)	5804.6(14)	29.4(4)
C19	5535.2(19)	7254.6(17)	6761.5(14)	31.0(4)
C16	3599.6(19)	9189.9(17)	6559.8(14)	29.5(4)
C23	5313.4(19)	6255.8(18)	8172.4(15)	32.8(5)

Atom	*x*	*y*	*z*	U(eq)
C22	6530.7(19)	6066.1(18)	8514.1(15)	34.4(5)
C27	-3896.5(19)	7096.5(18)	3524.9(14)	32.6(5)
C26	-3631(2)	3940.2(18)	5553.5(16)	35.7(5)
C25	9010(2)	5704(2)	9113.1(16)	39.0(5)

Table S7. Anisotropic Displacement Parameters ($Å^2 \times 10^3$) for **8a**. The Anisotropic displacement factor exponent takes the form: $-2\pi^2[h^2a^{*2}U_{11}+2hka^*b^*U_{12}+\cdots]$

Atom	U_{11}	U_{22}	U_{33}	U_{23}	U_{13}	U_{12}
O9	27.6(8)	22.8(6)	33.1(7)	3.2(5)	-2.0(6)	-0.3(5)
O28	28.3(8)	20.7(6)	37.1(8)	4.4(5)	0.6(6)	1.8(5)
O7	27.9(8)	24.0(6)	37.5(8)	4.0(5)	0.5(6)	-2.6(5)
O8	26.2(8)	25.5(6)	30.8(7)	1.0(5)	0.3(6)	3.5(5)
O17	33.6(8)	24.8(7)	42.0(8)	5.0(6)	2.0(7)	0.3(5)
O24	27.1(8)	39.7(8)	36.4(8)	4.9(6)	-0.2(6)	4.1(6)
C4	27.7(11)	24.3(9)	27.7(9)	0.3(7)	2.1(8)	2.2(7)
C2	26.2(11)	23.2(8)	28.6(9)	0.2(7)	0.6(8)	2.7(7)
C10	29.4(11)	21.8(8)	27.7(9)	-0.1(7)	2.0(8)	0.1(7)
C5	26.6(11)	24.6(9)	27.4(9)	0.8(7)	-0.3(8)	2.2(7)
C1	25.0(10)	25.8(9)	28.7(9)	-1.1(7)	1.8(8)	-0.5(7)
C15	32.2(11)	23.2(8)	29.5(9)	1.7(7)	1.9(8)	3.2(7)
C11	26.6(10)	24.5(9)	26.7(9)	-0.1(7)	1.1(8)	2.6(7)
C14	28.3(11)	26.0(9)	26.7(9)	-0.7(7)	2.4(8)	1.2(7)
C21	27.7(11)	26.3(9)	33.4(10)	-1.4(7)	1.7(8)	2.0(7)
C3	28.1(11)	22.6(8)	26.5(9)	1.0(7)	2.1(8)	1.4(7)
C13	28.5(10)	25.3(9)	26.8(9)	0.3(7)	1.6(8)	1.6(7)
C18	29.7(11)	22.8(8)	29.2(9)	-0.8(7)	1.1(8)	0.8(7)

Atom	U_{11}	U_{22}	U_{33}	U_{23}	U_{13}	U_{12}
C20	31.4(11)	31.9(10)	31.9(10)	2.9(8)	5.2(9)	0.1(8)
C12	30.3(11)	23.1(8)	27.4(9)	1.0(7)	2.5(8)	1.6(7)
C6	29.8(11)	23.3(9)	30.7(10)	1.5(7)	2.2(8)	-0.5(7)
C19	32.6(11)	27.7(9)	28.6(9)	2.0(7)	2.7(8)	3.2(8)
C16	27.7(11)	27.1(9)	29.5(10)	-1.2(7)	1.9(8)	0.7(7)
C23	30.5(11)	31.3(10)	32.9(10)	4.6(8)	3.6(8)	0.9(8)
C22	31.8(12)	33.1(10)	32.8(10)	5.5(8)	1.5(9)	3.6(8)
C27	31.9(11)	29.2(9)	30.8(10)	1.6(8)	0.3(8)	2.3(8)
C26	33.2(12)	25.4(9)	43.3(12)	5.2(8)	3.8(9)	-3.5(8)
C25	32.8(12)	34.7(11)	42.3(12)	6.8(9)	0.3(10)	5.5(9)

Table S8. Bond Lengths for **8a.**

Atom	Atom	Length/Å	Atom	Atom	Length/Å
O9	C10	1.370(2)	C10	C11	1.414(2)
O9	C5	1.392(2)	C5	C6	1.380(3)
O28	C3	1.356(2)	C1	C6	1.394(3)
O7	C1	1.360(2)	C15	C14	1.398(3)
O7	C26	1.433(2)	C11	C12	1.386(3)
O8	C2	1.386(2)	C14	C13	1.426(3)
O8	C27	1.437(2)	C14	C18	1.479(3)
O17	C16	1.219(2)	C21	C20	1.394(3)
O24	C21	1.369(2)	C21	C22	1.386(3)
O24	C25	1.425(2)	C13	C12	1.404(3)
C4	C5	1.391(3)	C13	C16	1.470(3)
C4	C11	1.445(3)	C18	C19	1.406(3)
C4	C3	1.404(3)	C18	C23	1.399(3)

Atom	Atom	Length/Å	Atom	Atom	Length/Å
C2	C1	1.412(3)	C20	C19	1.379(3)
C2	C3	1.385(3)	C23	C22	1.388(3)
C10	C15	1.371(3)			

Table S9. Bond Angles for **8a**.

Atom	Atom	Atom	Angle/°	Atom	Atom	Atom	Angle/°
C10	O9	C5	106.16(14)	C15	C14	C13	118.67(18)
C1	O7	C26	116.69(15)	C15	C14	C18	118.50(17)
C2	O8	C27	113.36(14)	C13	C14	C18	122.73(17)
C21	O24	C25	117.19(17)	O24	C21	C20	115.05(18)
C5	C4	C11	106.39(16)	O24	C21	C22	125.09(18)
C5	C4	C3	117.88(18)	C22	C21	C20	119.9(2)
C3	C4	C11	135.73(17)	O28	C3	C4	118.81(17)
O8	C2	C1	120.41(17)	O28	C3	C2	122.71(17)
C3	C2	O8	117.98(16)	C2	C3	C4	118.47(17)
C3	C2	C1	121.45(17)	C14	C13	C16	120.04(18)
O9	C10	C15	124.47(16)	C12	C13	C14	121.55(18)
O9	C10	C11	111.28(17)	C12	C13	C16	118.26(17)
C15	C10	C11	124.25(18)	C19	C18	C14	122.04(17)
C4	C5	O9	110.95(16)	C23	C18	C14	120.93(19)
C6	C5	O9	123.37(16)	C23	C18	C19	116.91(19)
C6	C5	C4	125.67(18)	C19	C20	C21	119.79(19)
O7	C1	C2	114.44(16)	C11	C12	C13	119.45(17)
O7	C1	C6	124.45(17)	C5	C6	C1	115.37(17)
C6	C1	C2	121.11(18)	C20	C19	C18	121.85(18)
C10	C15	C14	118.33(17)	O17	C16	C13	124.10(19)

Atom	Atom	Atom	Angle/°	Atom	Atom	Atom	Angle/°
C10	C11	C4	105.20(16)	C22	C23	C18	121.9(2)
C12	C11	C4	137.08(17)	C21	C22	C23	119.67(19)
C12	C11	C10	117.70(18)				

Table S10. Torsion Angles for **8a**.

A	B	C	D	Angle/°	A	B	C	D	Angle/°
O9	C10	C15	C14	179.30(17)	C11	C4	C5	O9	-1.2(2)
O9	C10	C11	C4	0.3(2)	C11	C4	C5	C6	178.07(19)
O9	C10	C11	C12	179.00(16)	C11	C4	C3	O28	-0.1(3)
O9	C5	C6	C1	179.83(17)	C11	C4	C3	C2	-178.9(2)
O7	C1	C6	C5	-178.30(17)	C11	C10	C15	C14	0.0(3)
O8	C2	C1	O7	2.2(3)	C14	C13	C12	C11	0.8(3)
O8	C2	C1	C6	-177.48(17)	C14	C13	C16	O17	174.11(19)
O8	C2	C3	O28	-2.4(3)	C14	C18	C19	C20	-177.20(18)
O8	C2	C3	C4	176.37(16)	C14	C18	C23	C22	176.11(18)
O24	C21	C20	C19	179.11(17)	C21	C20	C19	C18	1.8(3)
O24	C21	C22	C23	179.74(18)	C3	C4	C5	O9	178.93(16)
C4	C5	C6	C1	0.7(3)	C3	C4	C5	C6	-1.8(3)
C4	C11	C12	C13	179.2(2)	C3	C4	C11	C10	-179.6(2)
C2	C1	C6	C5	1.3(3)	C3	C4	C11	C12	2.1(4)
C10	O9	C5	C4	1.4(2)	C3	C2	C1	O7	177.48(17)
C10	O9	C5	C6	-177.90(18)	C3	C2	C1	C6	-2.2(3)
C10	C15	C14	C13	2.0(3)	C13	C14	C18	C19	-46.2(3)
C10	C15	C14	C18	-174.56(18)	C13	C14	C18	C23	138.0(2)
C10	C11	C12	C13	1.1(3)	C18	C14	C13	C12	173.97(18)
C5	O9	C10	C15	179.54(18)	C18	C14	C13	C16	-10.7(3)

A	B	C	D	Angle/°	A	B	C	D	Angle/°
C5	O9	C10	C11	-1.0(2)	C18	C23	C22	C21	0.4(3)
C5	C4	C11	C10	0.5(2)	C20	C21	C22	C23	0.2(3)
C5	C4	C11	C12	-177.8(2)	C12	C13	C16	O17	-10.4(3)
C5	C4	C3	O28	179.74(16)	C19	C18	C23	C22	0.1(3)
C5	C4	C3	C2	0.9(3)	C16	C13	C12	C11	-174.61(17)
C1	C2	C3	O28	-177.80(17)	C23	C18	C19	C20	-1.2(3)
C1	C2	C3	C4	1.0(3)	C22	C21	C20	C19	-1.3(3)
C15	C10	C11	C4	179.75(18)	C27	O8	C2	C1	-81.7(2)
C15	C10	C11	C12	-1.6(3)	C27	O8	C2	C3	102.85(19)
C15	C14	C13	C12	-2.5(3)	C26	O7	C1	C2	-169.70(17)
C15	C14	C13	C16	172.91(18)	C26	O7	C1	C6	10.0(3)
C15	C14	C18	C19	130.2(2)	C25	O24	C21	C20	-177.96(17)
C15	C14	C18	C23	-45.6(3)	C25	O24	C21	C22	2.5(3)

Table S11. Hydrogen Atom Coordinates (Å × 10^4) and Isotropic Displacement Parameters (Å^2 × 10^3) for **8a**.

**Atom**	***x***	***y***	***z***	**U(eq)**
H28	-1938.07	8800.31	4609.65	46
H15	3120.26	5225.78	7220.6	36
H20	7239.96	7392.54	6735.83	39
H12	1312.27	8861.73	5759.84	34
H6	-1379.69	4118.15	6012.06	35
H19	5200.37	7649.78	6153.6	37
H16	4407.01	9176.77	6949.7	35
H23	4828.3	5971.93	8548.11	39
H22	6869.01	5652.82	9113.17	41

**Atom**	***x***	***y***	***z***	**U(eq)**
H27A	-3280.1	7204.05	3202.26	49
H27B	-4563.57	7639.65	3227.79	49
H27C	-4173.95	6255.04	3449.4	49
H26A	-3202.59	3313.83	5311.08	53
H26B	-4471.43	3718.5	5392.34	53
H26C	-3293.06	4010.92	6262.91	53
H25A	8708.14	4871.3	9049.08	58
H25B	8829.46	6100.02	9662.48	58
H25C	9871.49	5692.36	9229.39	58

6. Copies of NMR spectra of intermediates

Figure S7. ^{1}H NMR spectrum of compound **2**

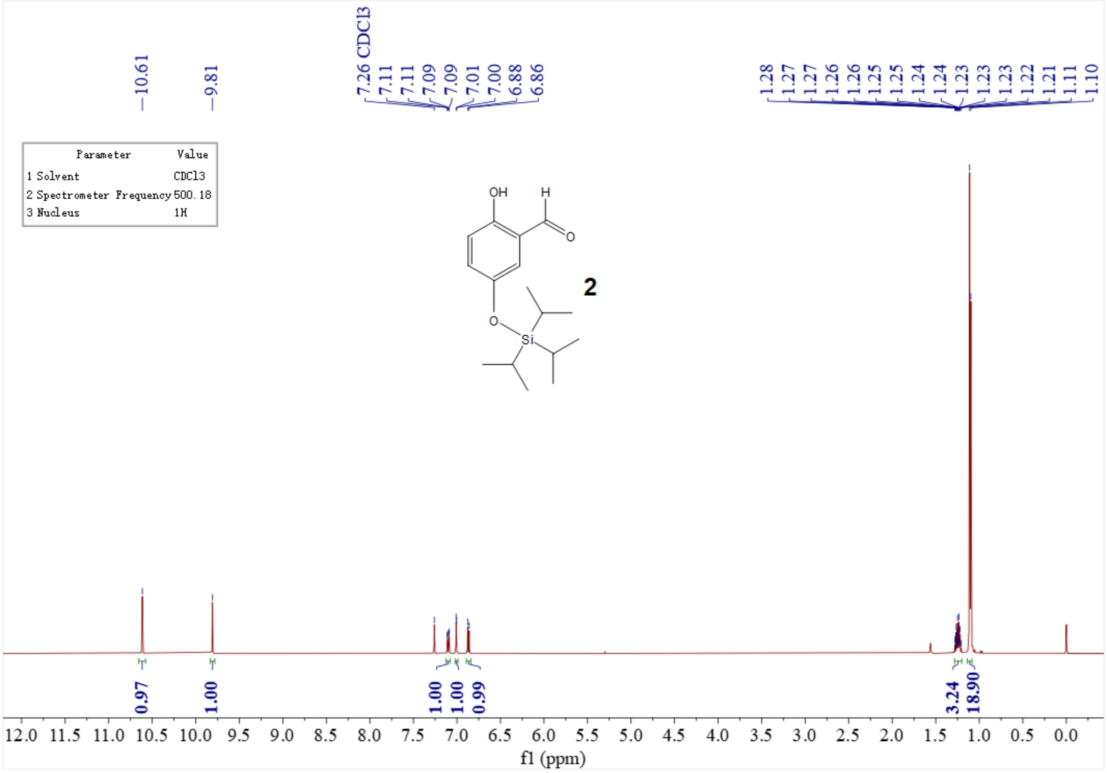

Figure S8. ^{13}C NMR spectrum of compound **2**

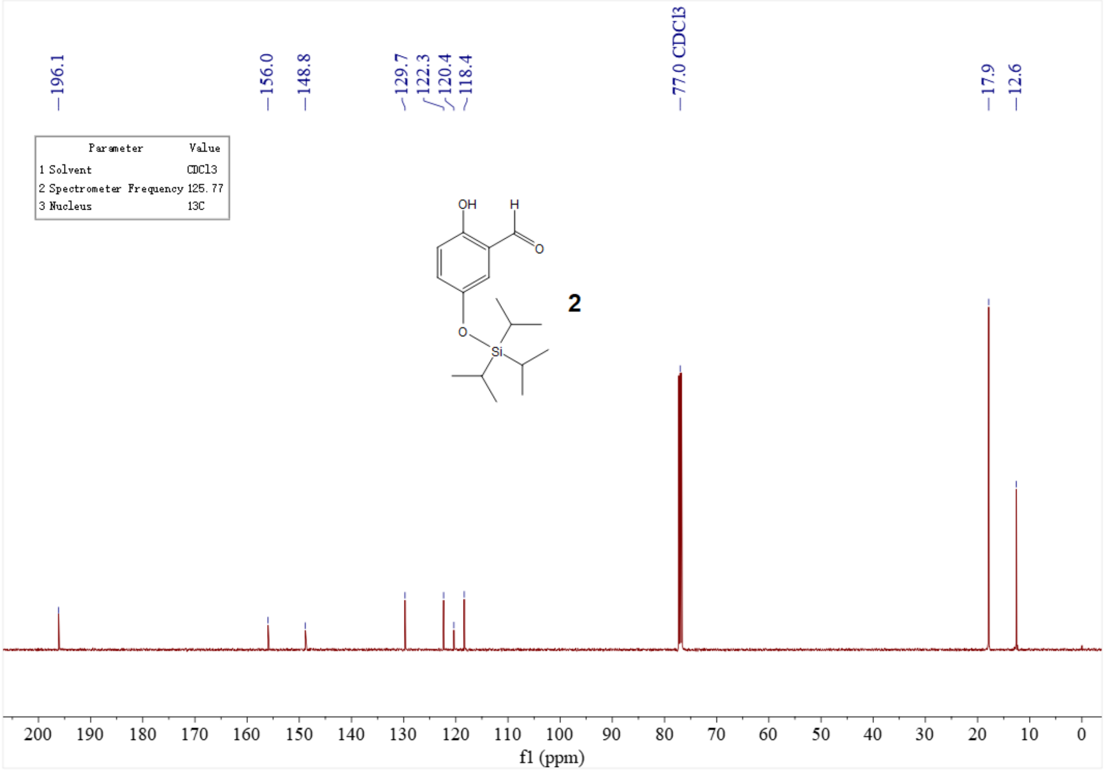

Figure S9. ^{1}H NMR spectrum of compound **3**

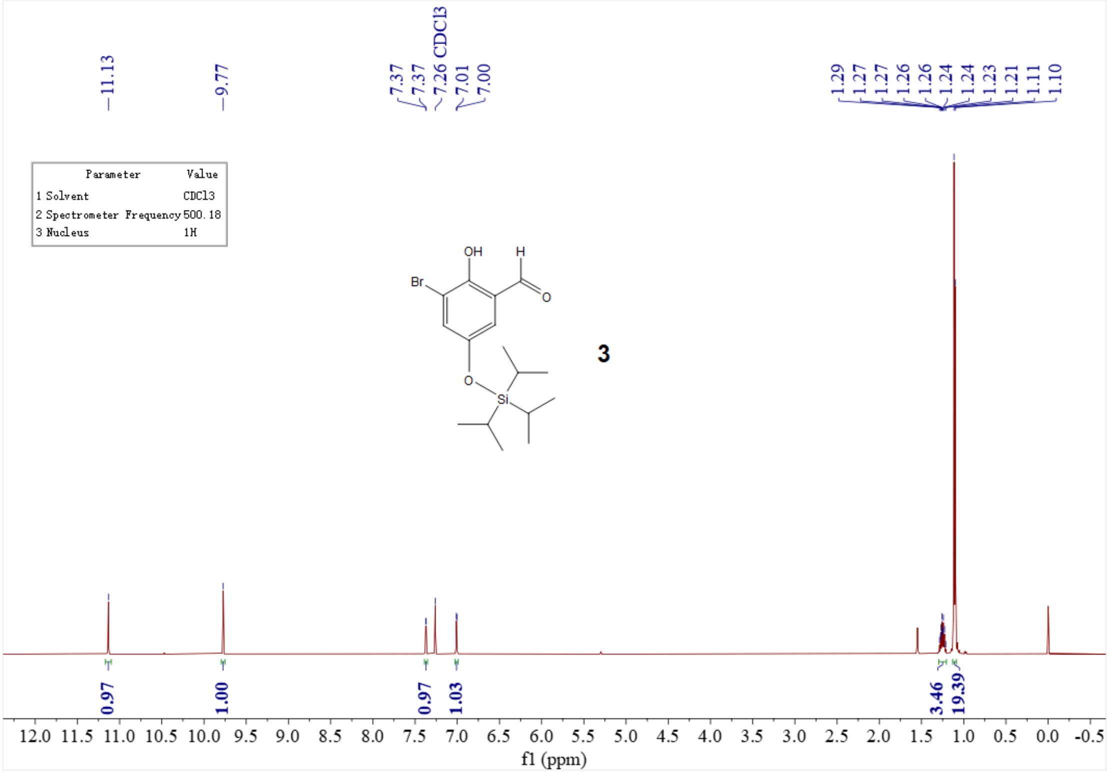

Figure S10. ^{13}C NMR spectrum of compound **3**

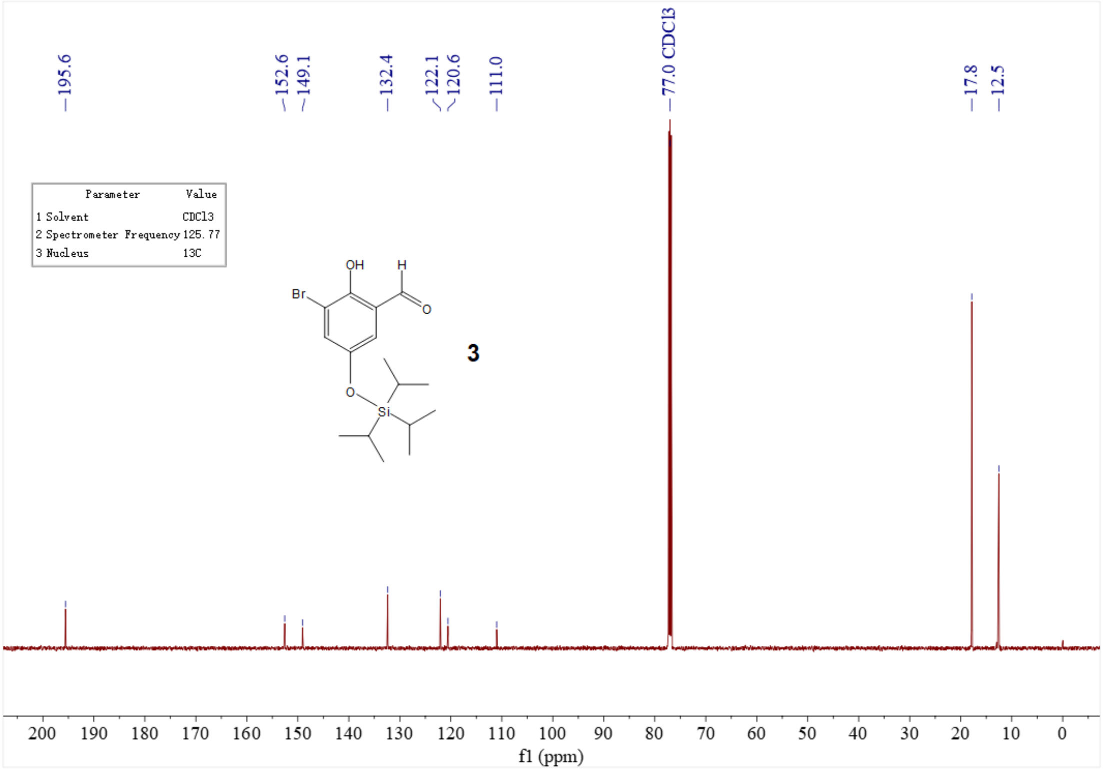

Figure S11. ^{1}H NMR spectrum of compound **4**

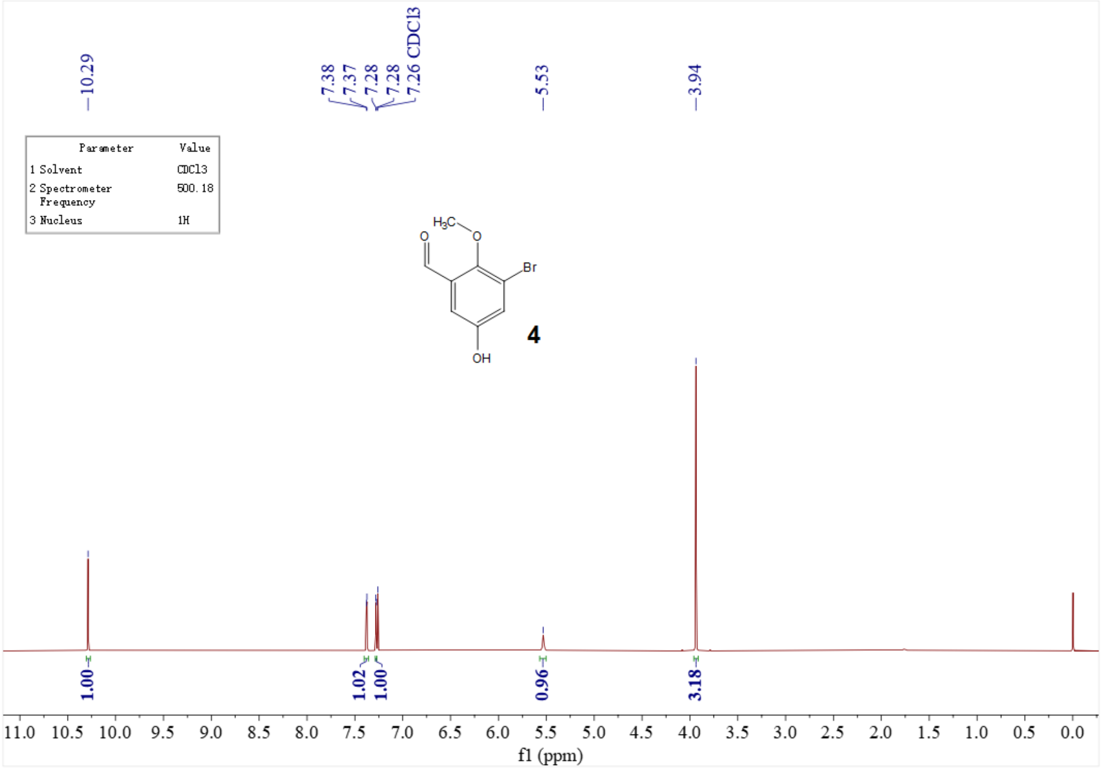

Figure S12. ^{13}C NMR spectrum of compound **4**

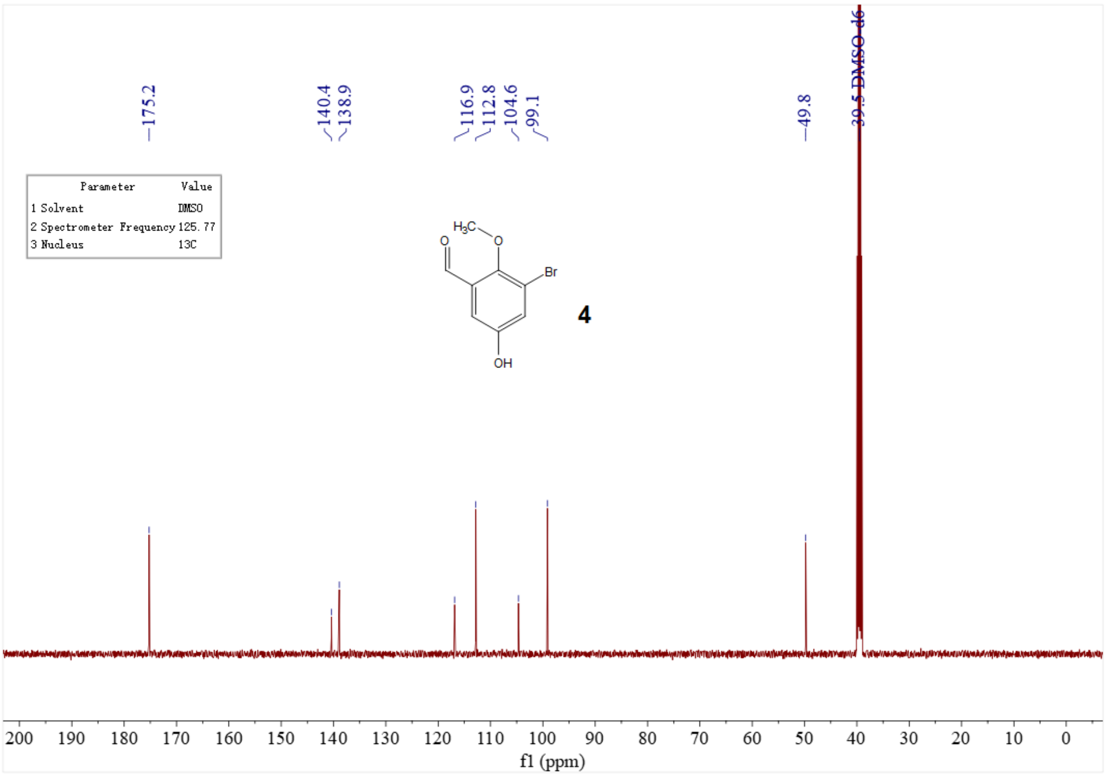

Figure S13. ^{1}H NMR spectrum of compound **6**

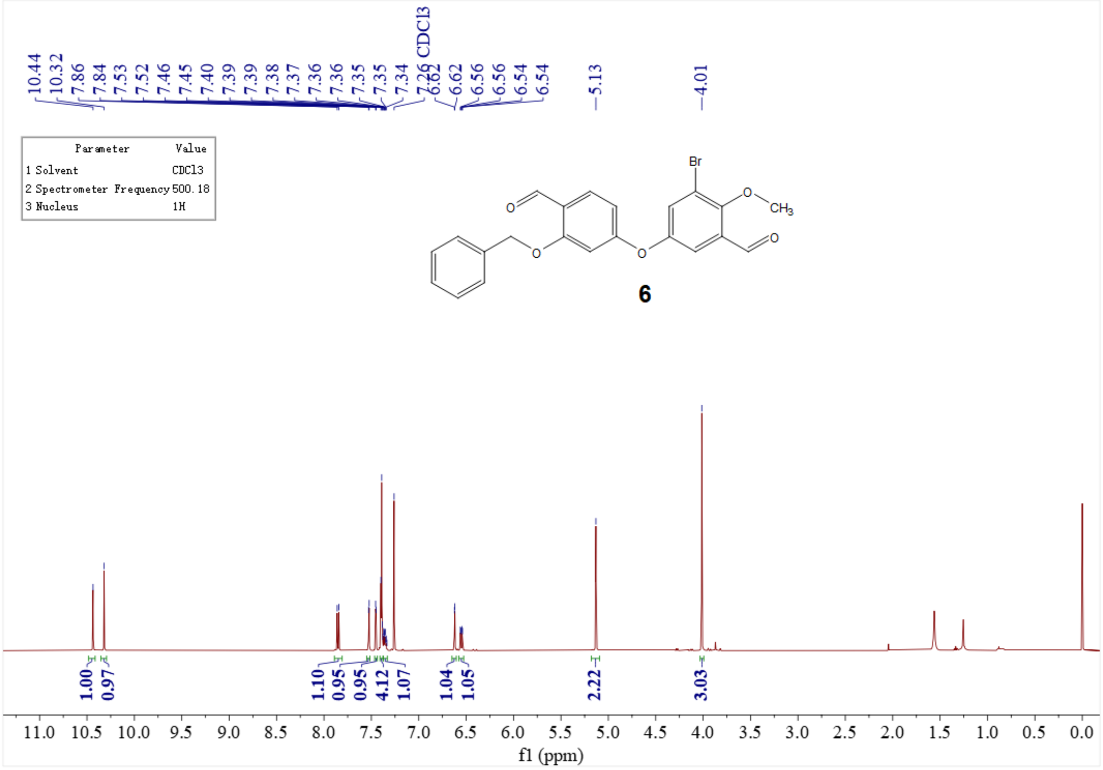

Figure S14. ^{13}C NMR spectrum of compound **6**

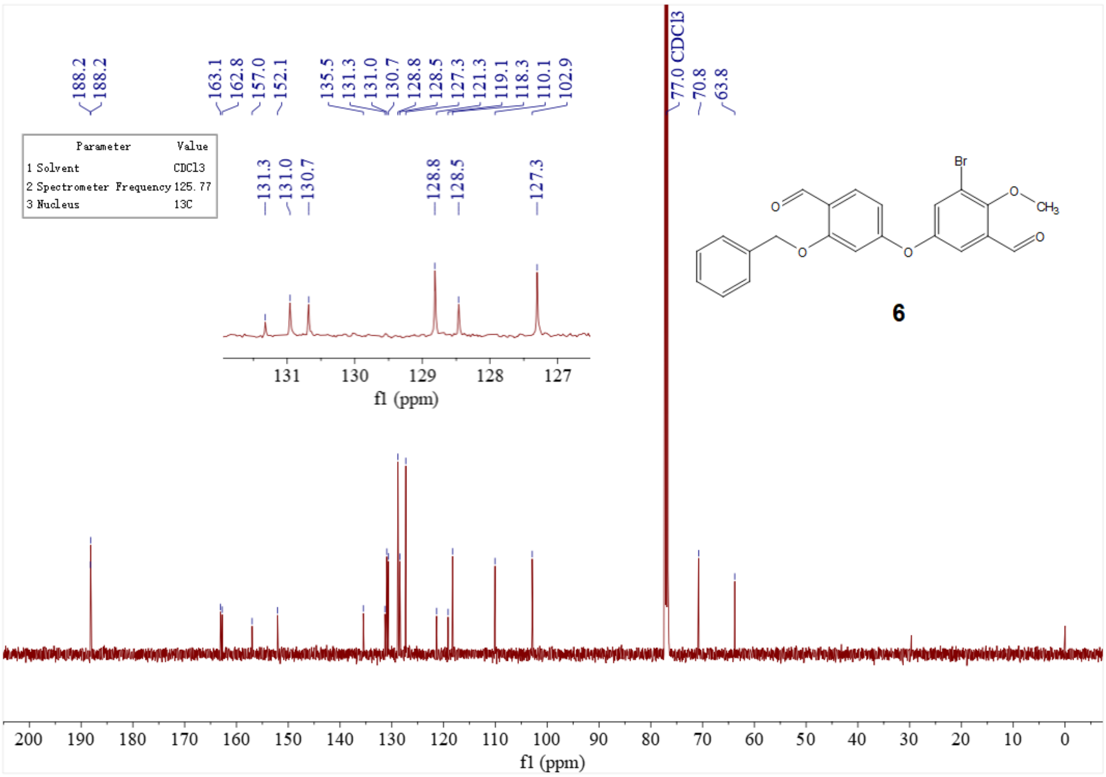

Figure S15. ^{1}H NMR spectrum of compound **8**

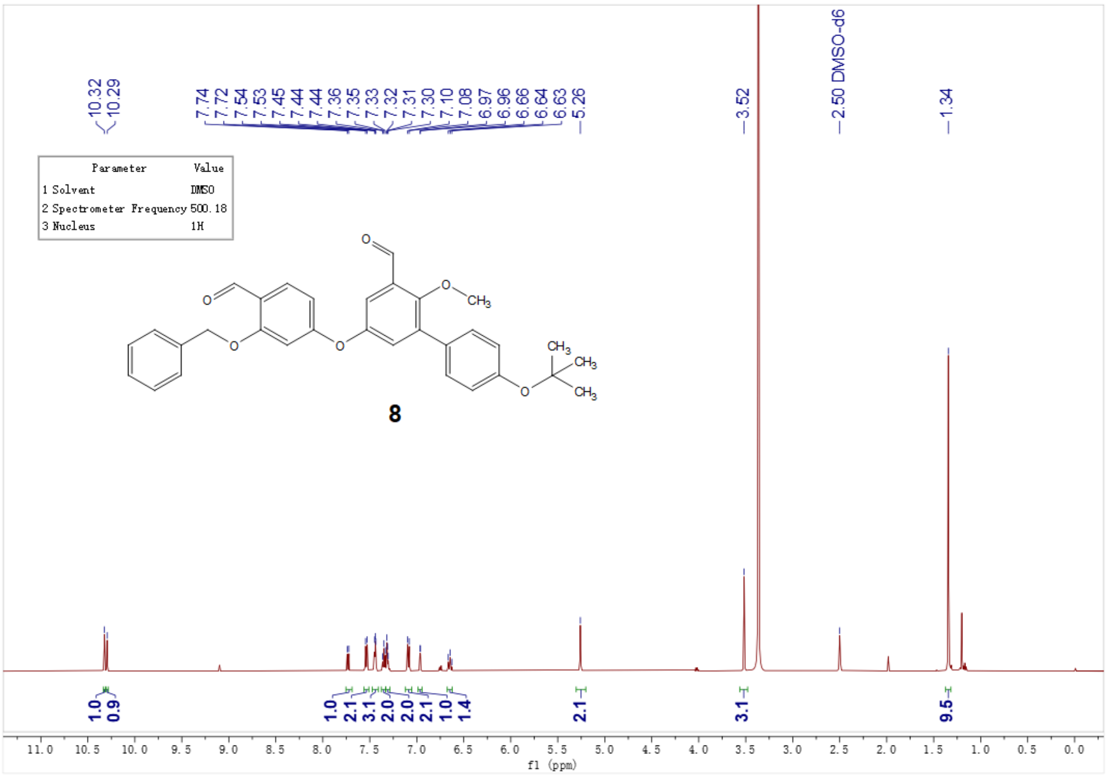

Figure S16. ^{13}C NMR spectrum of compound **8**

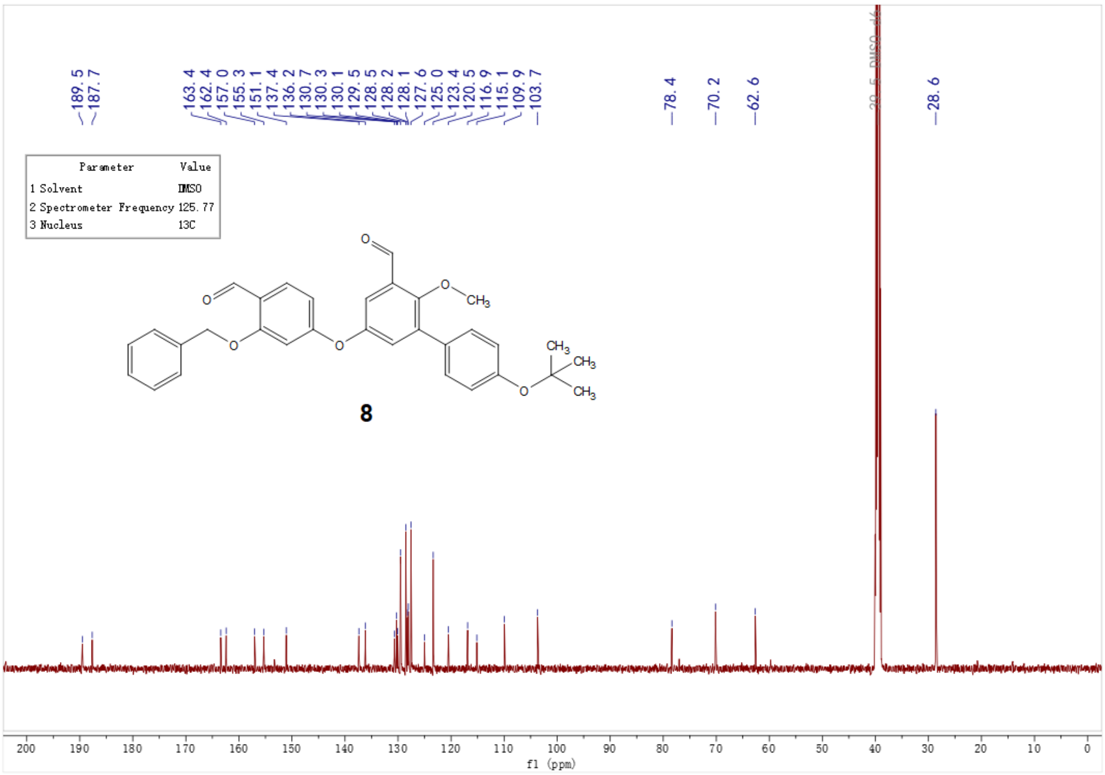

Figure S17. ^{1}H NMR spectrum of compound **8a**

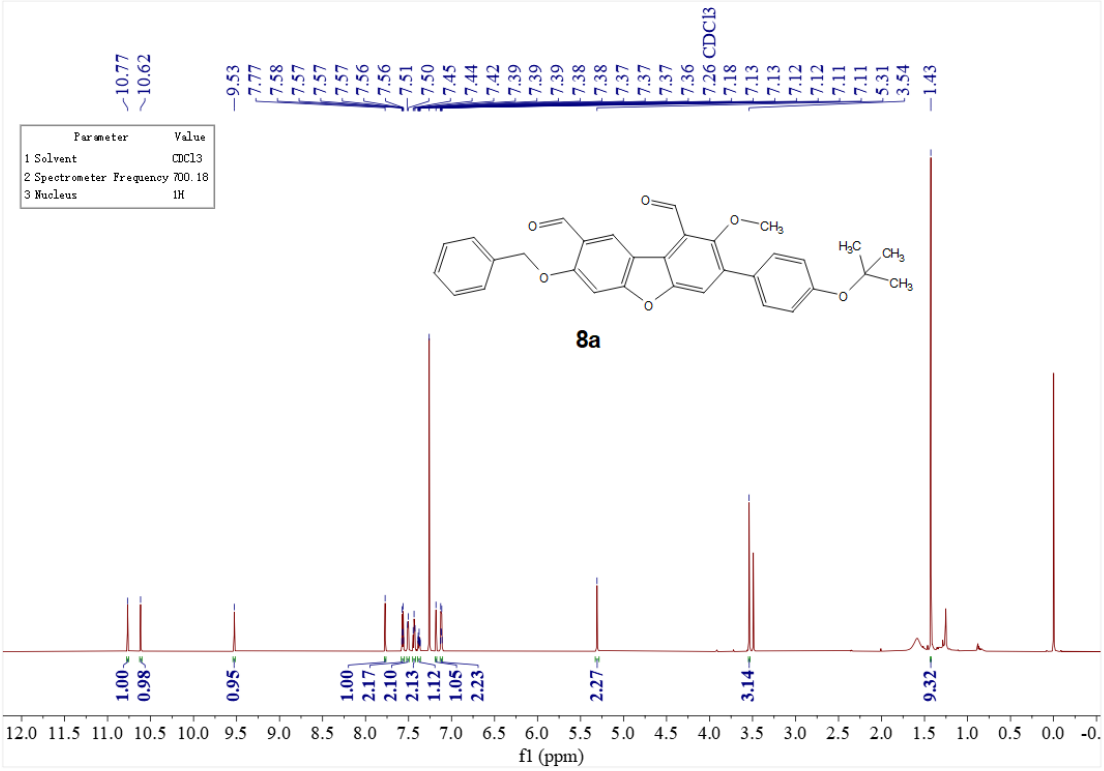

Figure S18. ^{13}C NMR spectrum of compound **8a**

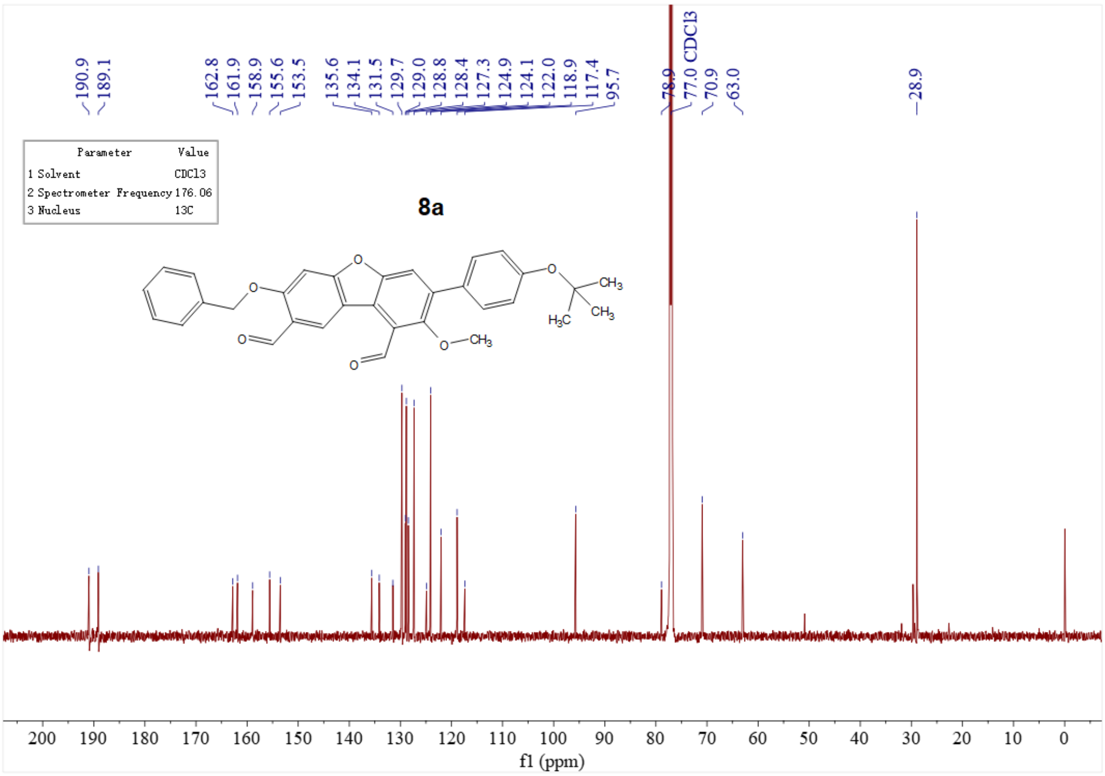

Figure S19. ^{1}H NMR spectrum of compound **9**

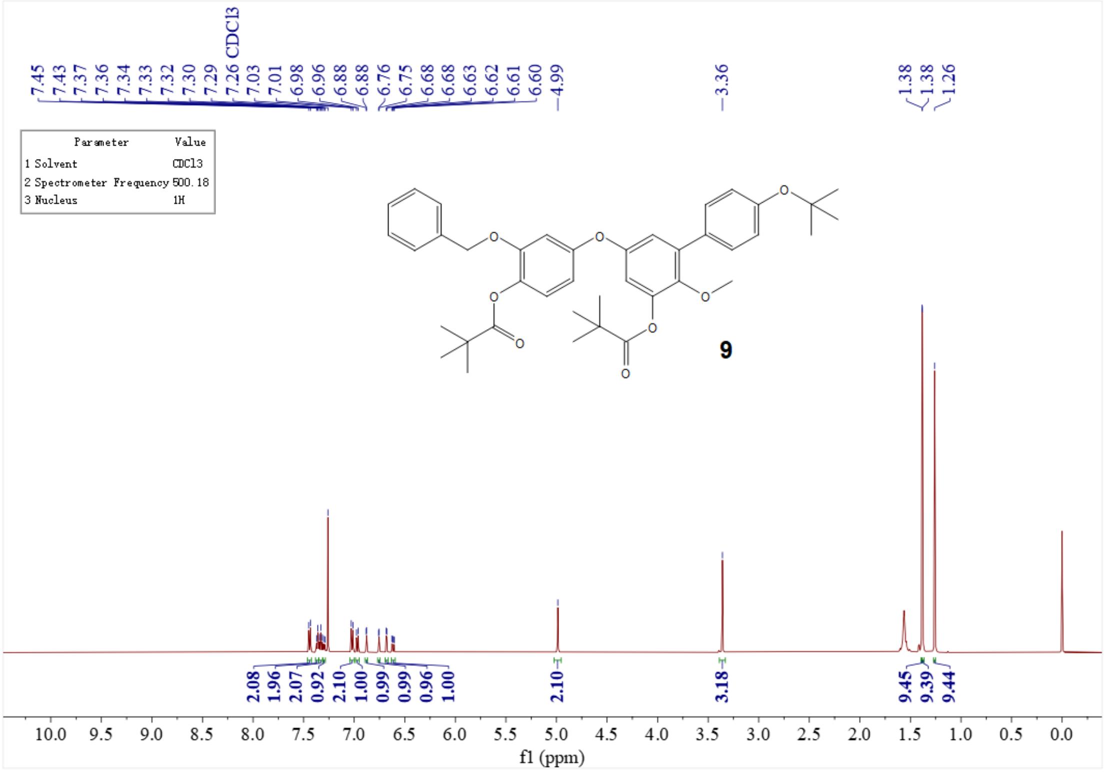

Figure S20. ^{13}C NMR spectrum of compound **9**

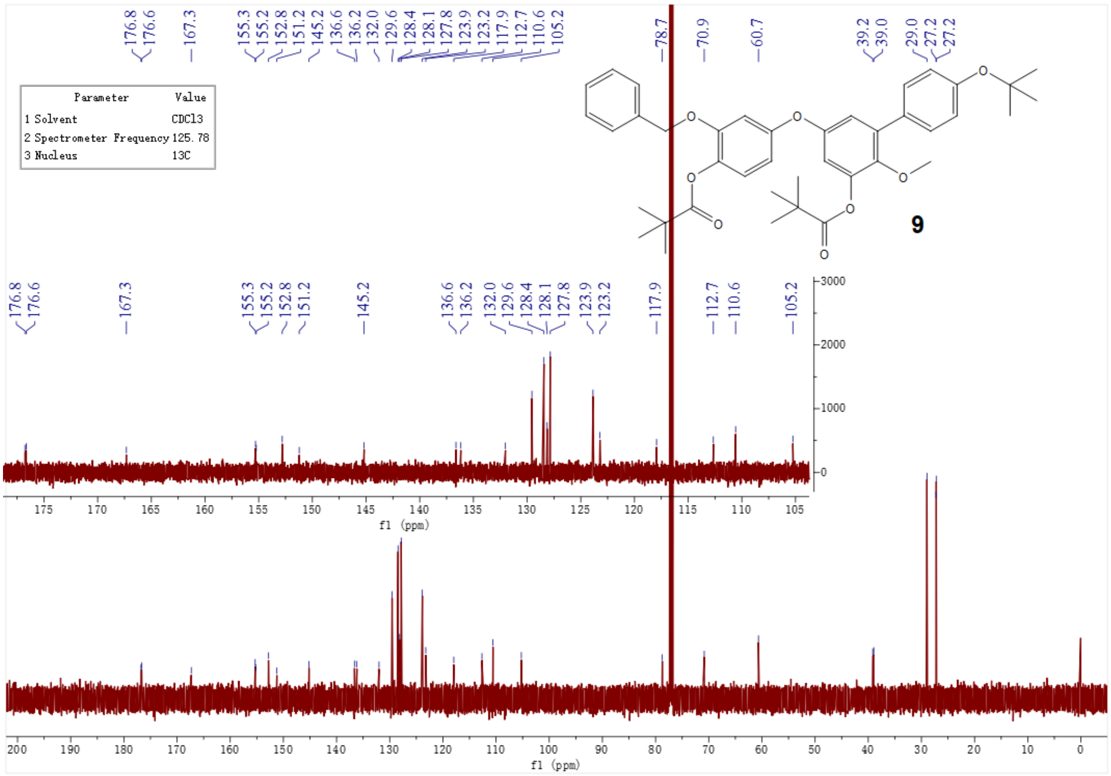

Figure S21. ^{1}H NMR spectrum of compound **9a**

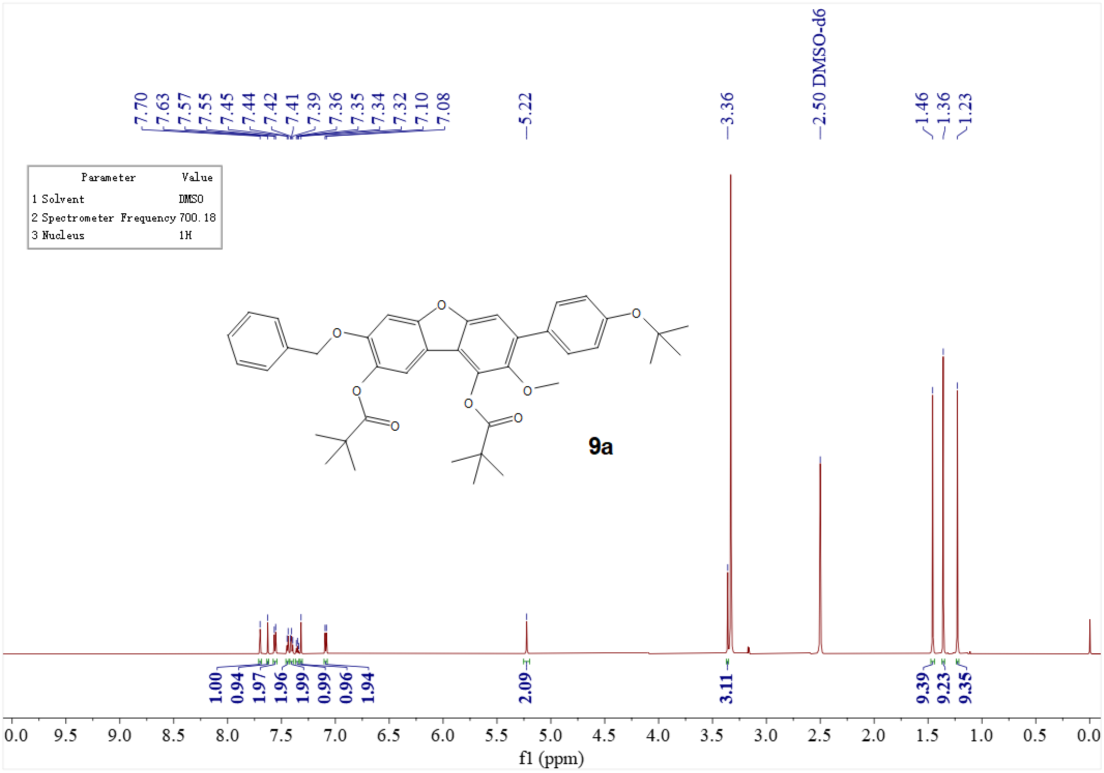

Figure S22. ^{13}C NMR spectrum of compound **9a**

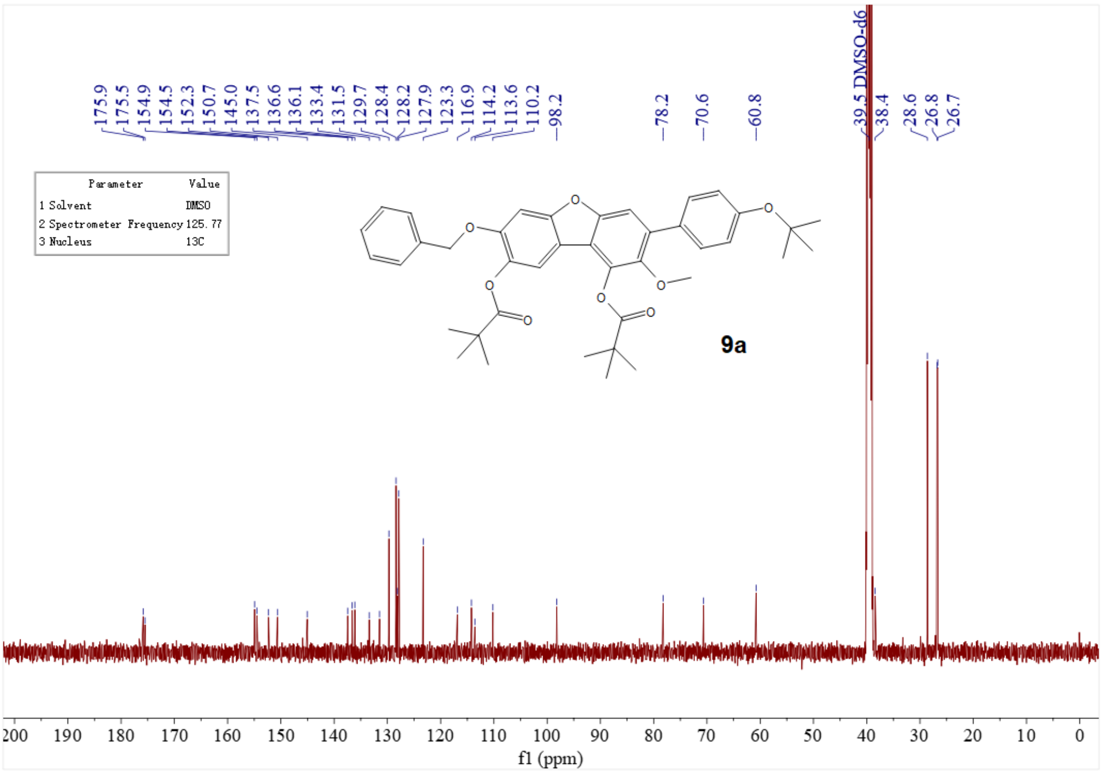

Figure S23. ^{1}H NMR spectrum of compound **10**

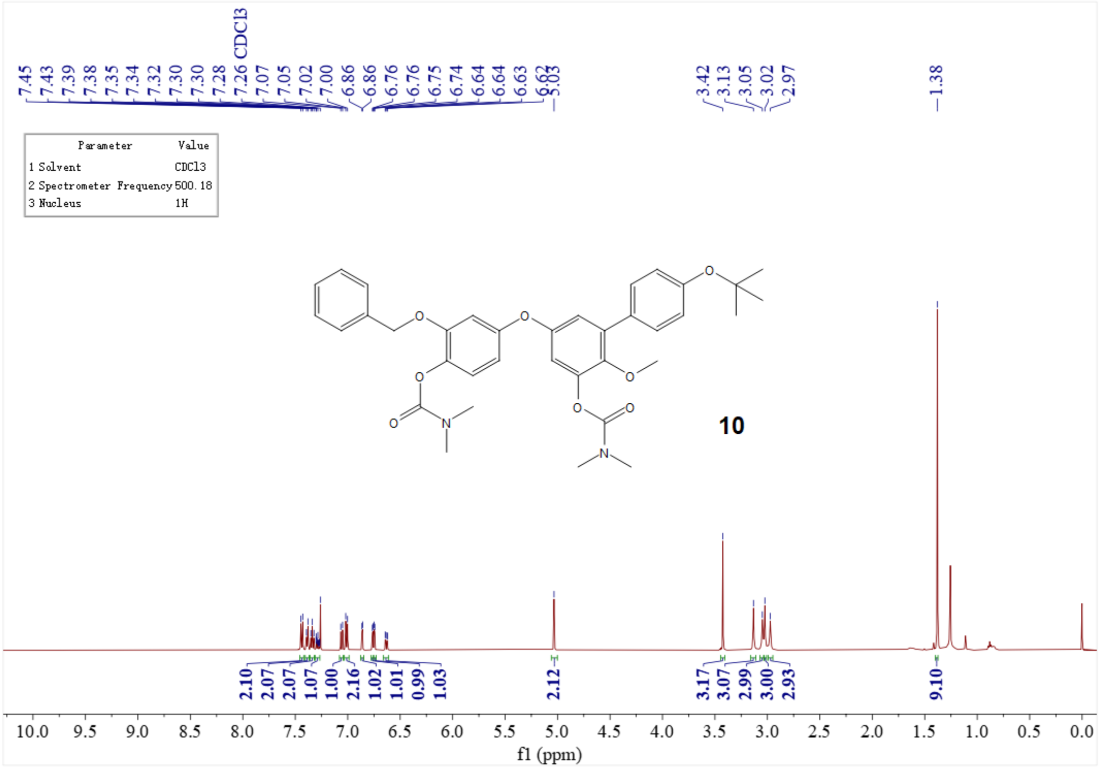

Figure S24. ^{13}C NMR spectrum of compound **10**

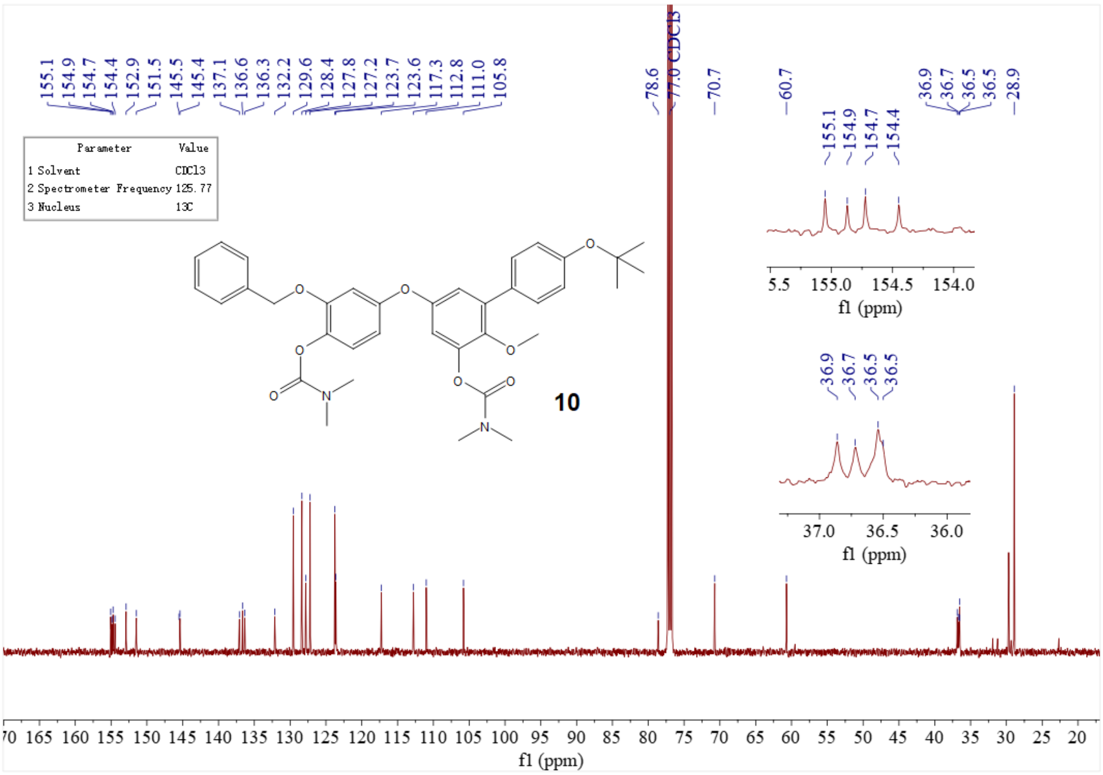

Figure S25. ^{1}H NMR spectrum of compound **10a**

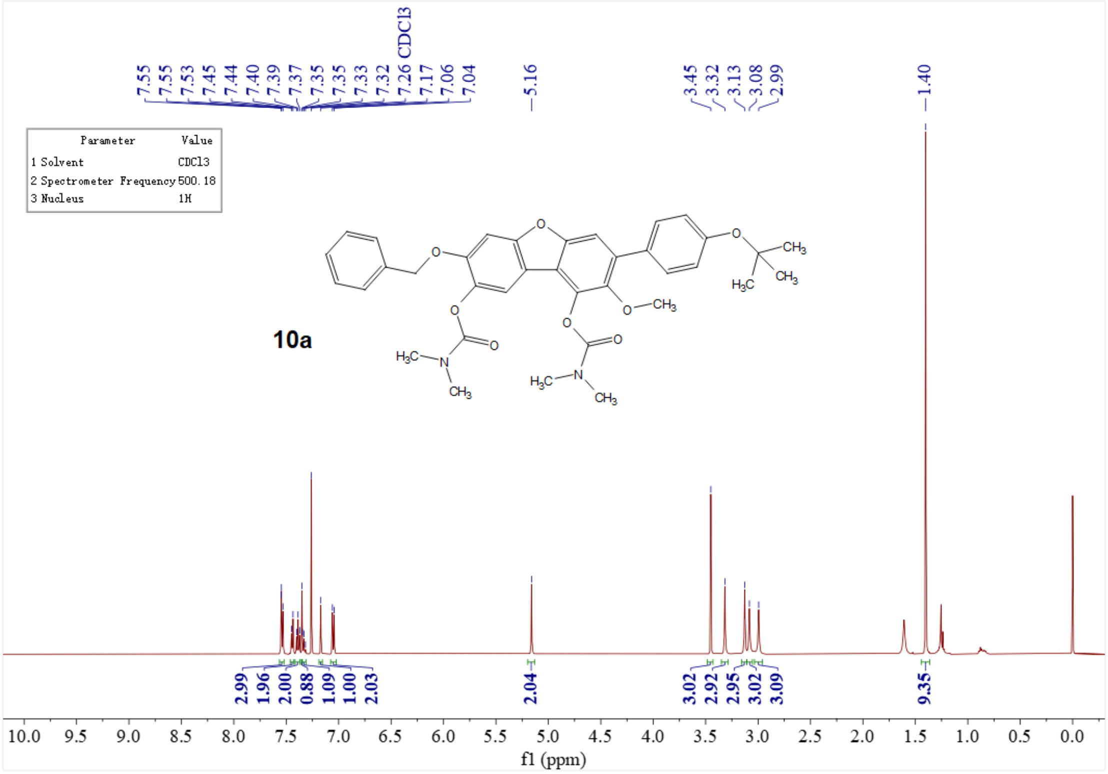

Figure S26. ^{13}C NMR spectrum of compound **10a**

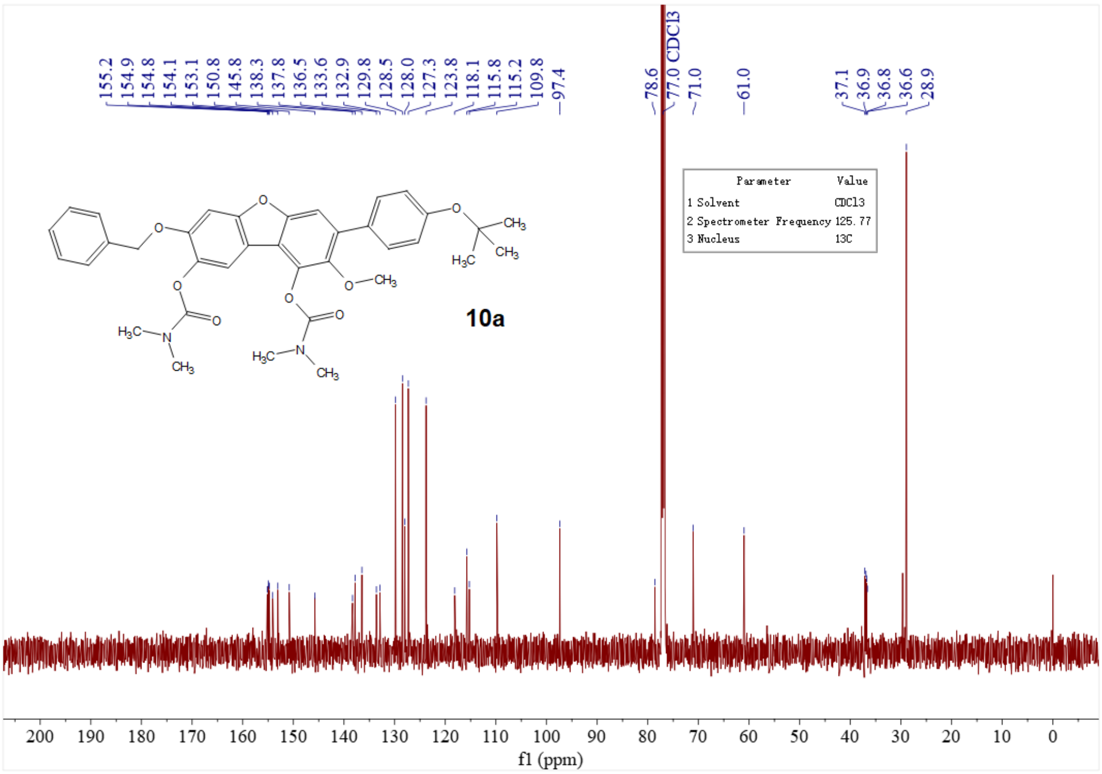

Figure S27. ^{1}H NMR spectrum of compound **11**

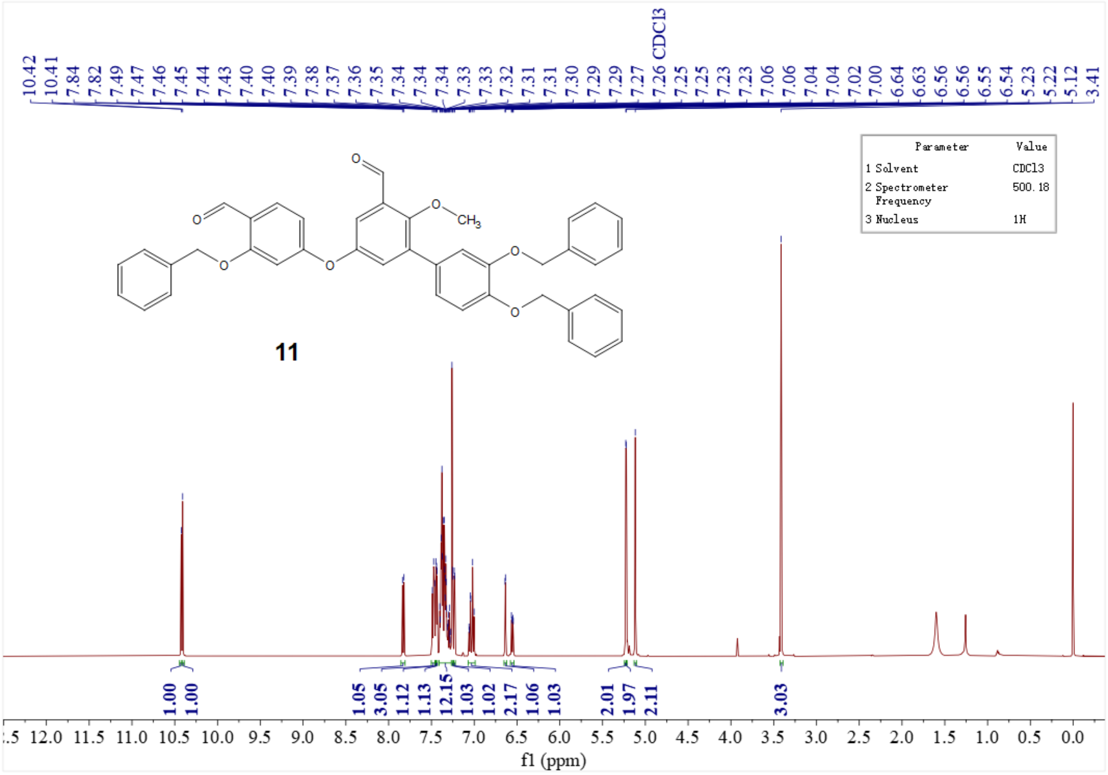

Figure S28. ^{13}C NMR spectrum of compound **11**

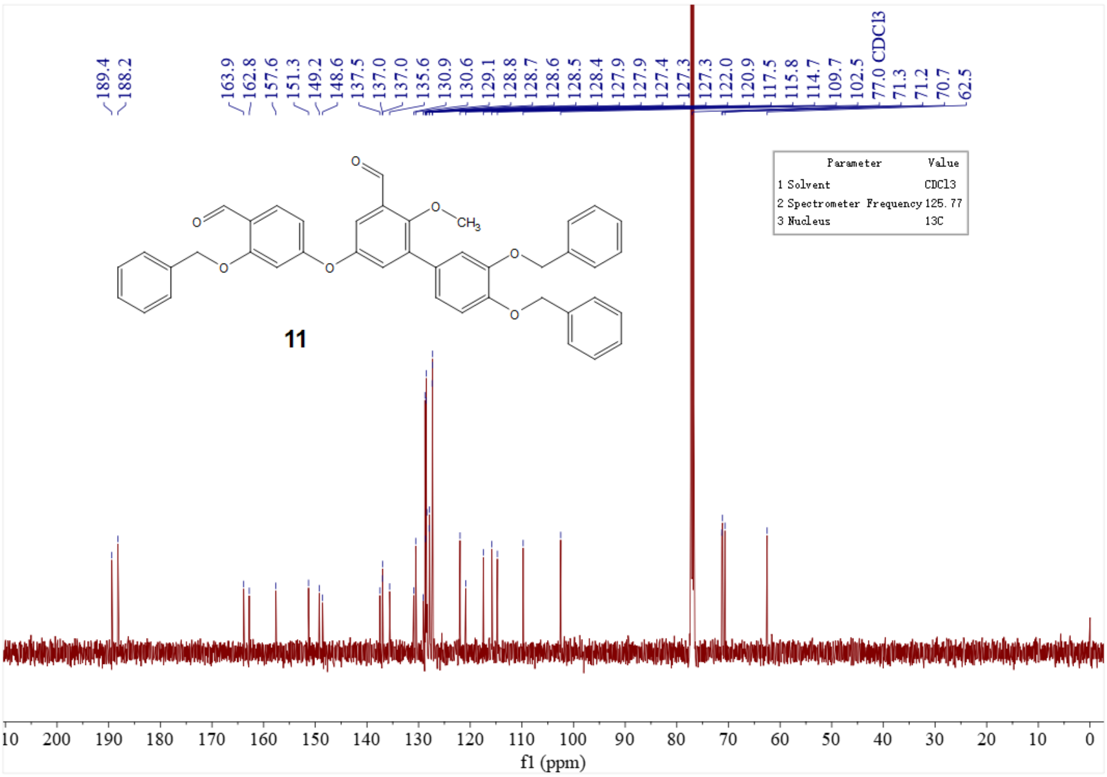

Figure S29. ^{1}H NMR spectrum of compound **12**

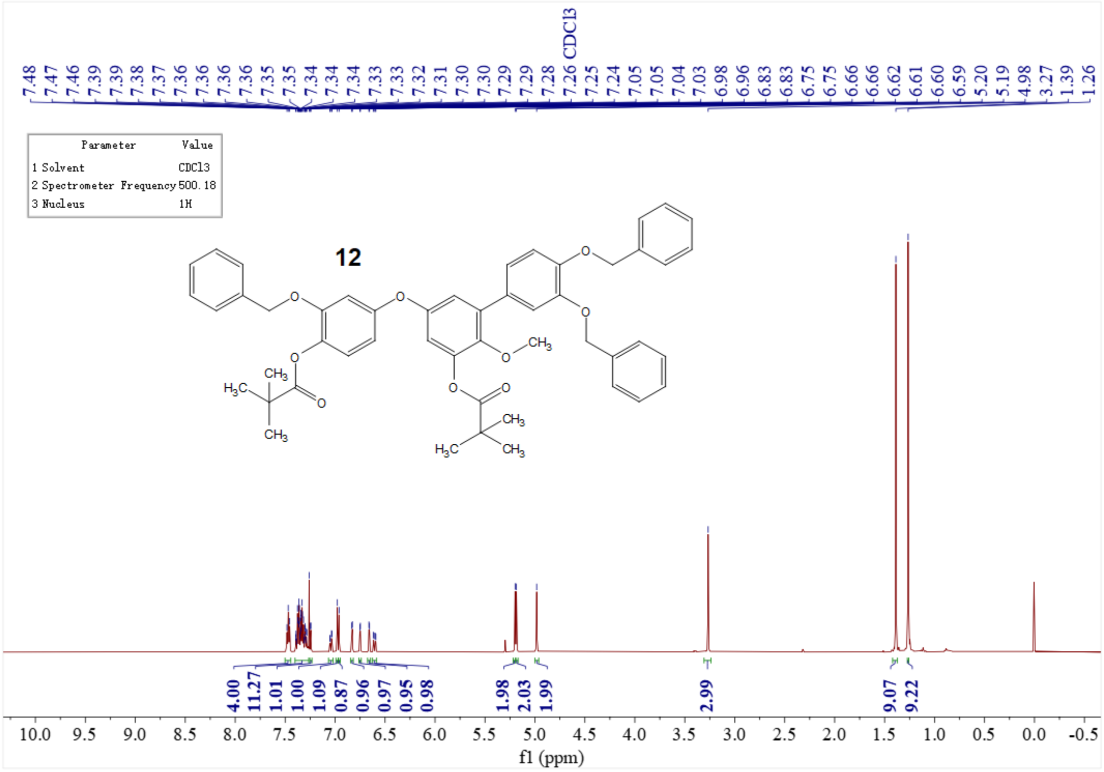

Figure S30. ^{13}C NMR spectrum of compound **12**

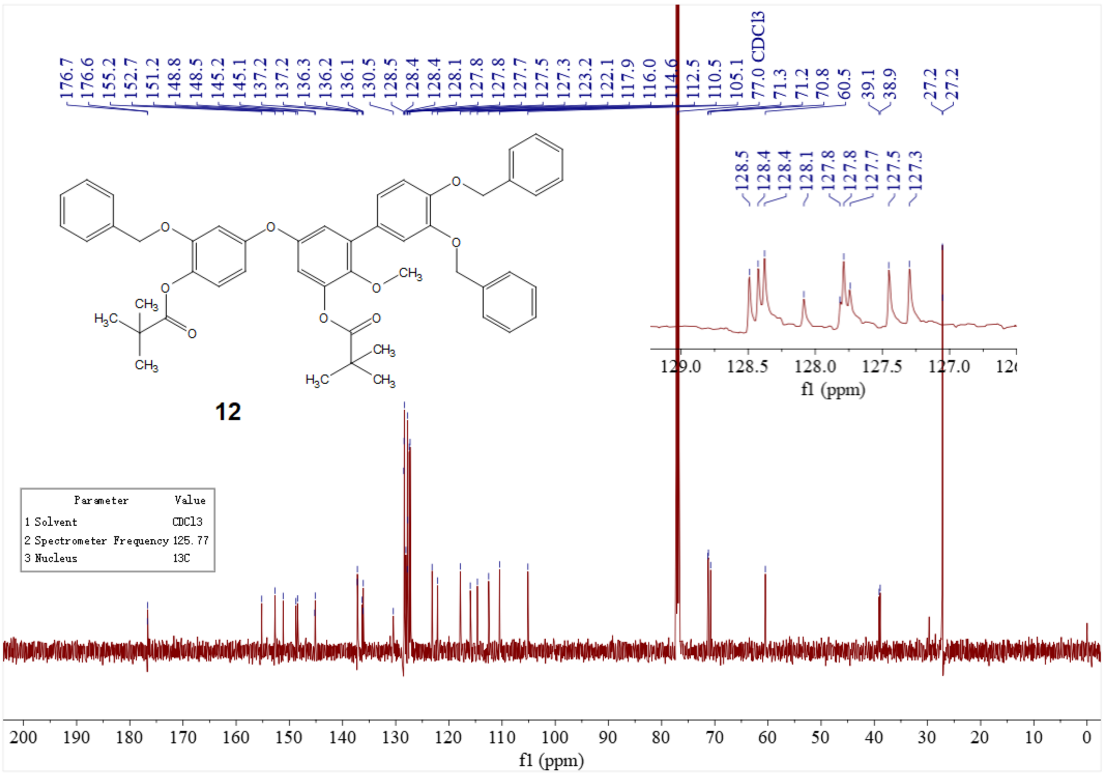

Figure S31. ^{1}H NMR spectrum of compound **12a**

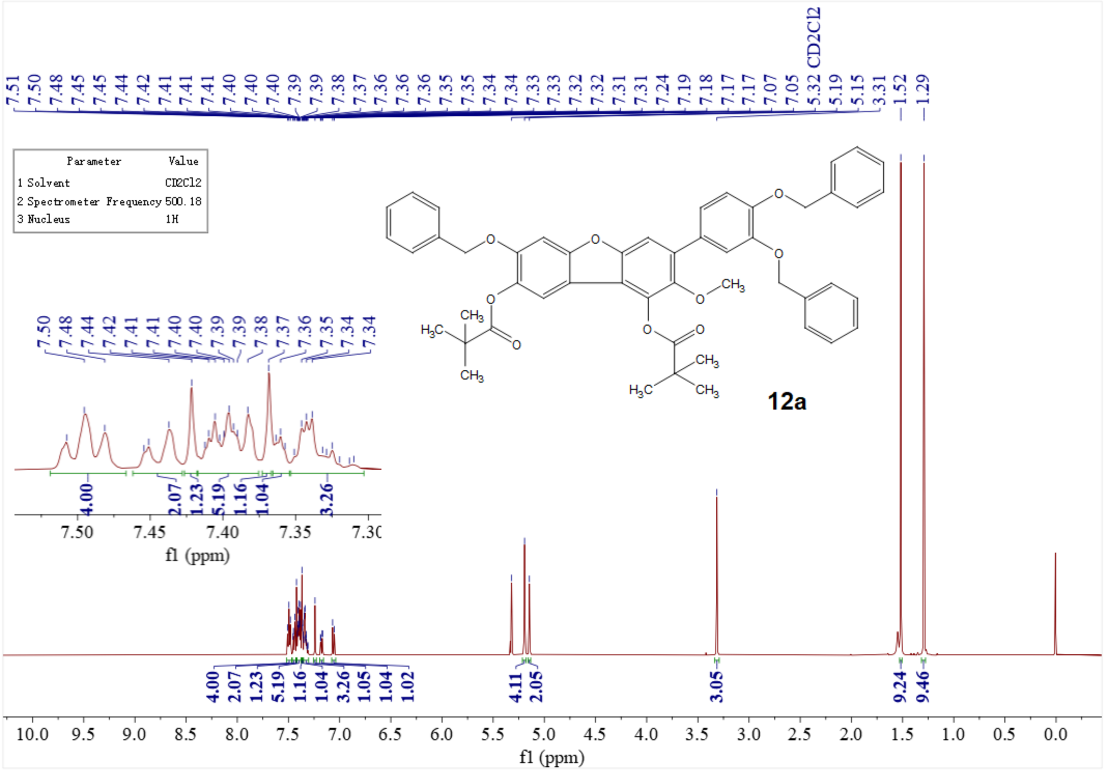

Figure S32. ^{13}C NMR spectrum of compound **12a**

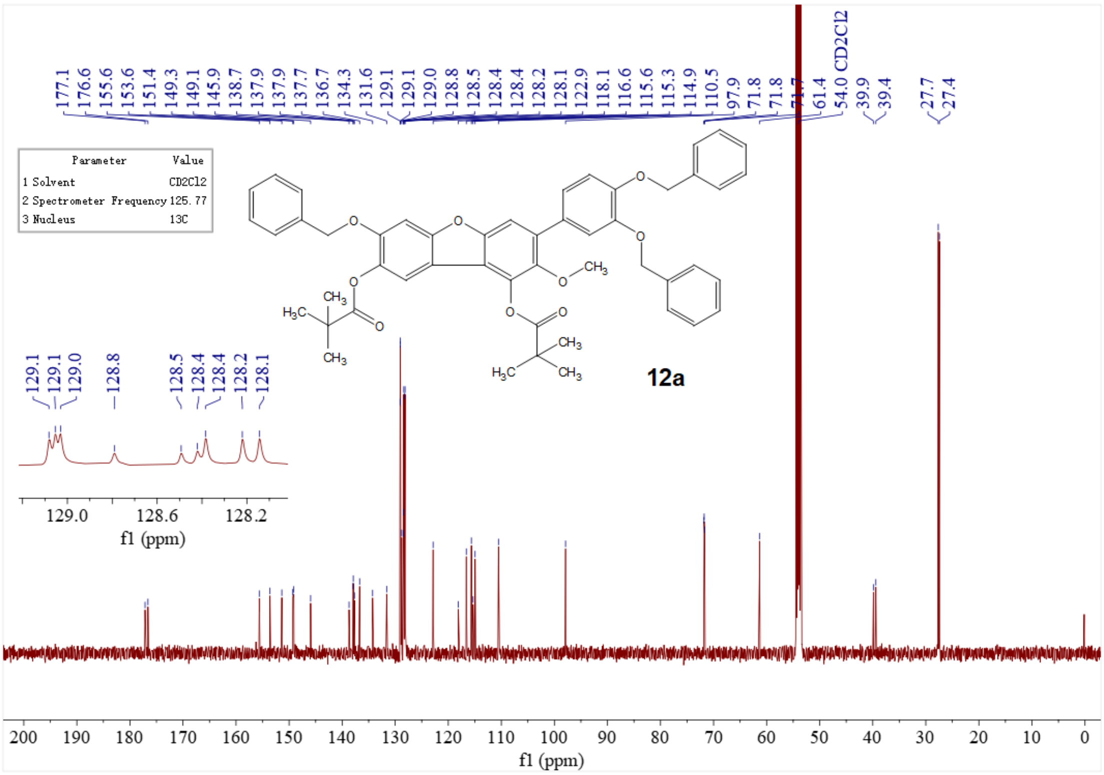

Supplement: Supplementary file 1 [file marinedrugs-23-00437-s001.zip › marinedrugs-3967332-supplementary-update.pdf]
